# Subdominant Outer Membrane Antigens in *Anaplasma marginale*: Conservation, Antigenicity, and Protective Capacity Using Recombinant Protein

**DOI:** 10.1371/journal.pone.0129309

**Published:** 2015-06-16

**Authors:** Deirdre R. Ducken, Wendy C. Brown, Debra C. Alperin, Kelly A. Brayton, Kathryn E. Reif, Joshua E. Turse, Guy H. Palmer, Susan M. Noh

**Affiliations:** 1 Animal Disease Research Unit, Agricultural Research Service, U. S. Department of Agriculture, Pullman, Washington, United States of America; 2 Program in Vector-Borne Diseases, Department of Veterinary Microbiology and Pathology, Washington State University, Pullman, Washington, United States of America; 3 Paul G. Allen School for Global Animal Health, Washington State University, Pullman, Washington, United States of America; Kansas State University, UNITED STATES

## Abstract

*Anaplasma marginale* is a tick-borne rickettsial pathogen of cattle with a worldwide distribution. Currently a safe and efficacious vaccine is unavailable. Outer membrane protein (OMP) extracts or a defined surface protein complex reproducibly induce protective immunity. However, there are several knowledge gaps limiting progress in vaccine development. First, are these OMPs conserved among the diversity of *A*. *marginale* strains circulating in endemic regions? Second, are the most highly conserved outer membrane proteins in the immunogens recognized by immunized and protected animals? Lastly, can this subset of OMPs recognized by antibody from protected vaccinates and conserved among strains recapitulate the protection of outer membrane vaccines? To address the first goal, genes encoding OMPs AM202, AM368, AM854, AM936, AM1041, and AM1096, major subdominant components of the outer membrane, were cloned and sequenced from geographically diverse strains and isolates. AM202, AM936, AM854, and AM1096 share 99.9 to 100% amino acid identity. AM1041 has 97.1 to 100% and AM368 has 98.3 to 99.9% amino acid identity. While all four of the most highly conserved OMPs were recognized by IgG from animals immunized with outer membranes, linked surface protein complexes, or unlinked surface protein complexes and shown to be protected from challenge, the highest titers and consistent recognition among vaccinates were to AM854 and AM936. Consequently, animals were immunized with recombinant AM854 and AM936 and challenged. Recombinant vaccinates and purified outer membrane vaccinates had similar IgG and IgG2 responses to both proteins. However, the recombinant vaccinates developed higher bacteremia after challenge as compared to adjuvant-only controls and outer membrane vaccinates. These results provide the first evidence that vaccination with specific antigens may exacerbate disease. Progressing from the protective capacity of outer membrane formulations to recombinant vaccines requires testing of additional antigens, optimization of the vaccine formulation and a better understanding of the protective immune response.

## Introduction

Diseases of both humans and animals caused by vector-borne pathogens that establish long-term persistent infection, such as malaria, theileriosis, babesiosis, ehrlichiosis and anaplasmosis are notoriously difficult to prevent using vaccination. In many cases, there is a strong precedence for the development of effective immunity particularly in response to infection with attenuated organisms. Genome sequencing and bioinformatics have provided comprehensive sets of surface proteins and potential targets of the protective immune response. However, identification of the specific proteins or groups of proteins required to induce protective immunity remains a challenge. In the absence of strong correlates of immunity, which is a common limitation in efforts toward vaccine development targeting these pathogens, prioritizing surface proteins for live animal testing is difficult.

Bovine anaplasmosis, caused by *Anaplasma marginale*, an obligate-intracellular rickettsial pathogen, is one of the most prevalent tick-borne diseases of cattle worldwide. Protective immunity to anaplasmosis can be induced by inoculation with live *A*. *marginale* subspecies *centrale*, or immunization using purified outer membranes or surface protein complexes [1–6]. While indicative that vaccine development based on outer membrane proteins is achievable, the outer membrane preparation contains over 20 proteins and is difficult to prepare and standardize. The long- term goal is to develop a recombinant vaccine through the identification of proteins within the outer membrane that are protective.

Within the protection-inducing outer membrane and surface protein complexes, two outer membrane proteins (OMPs), Major Surface Protein (MSP) 2 and MSP3, are highly abundant and immunodominant. However, both MSP2 and 3 undergo sequential antigenic variation during infection and there is strong evidence that these proteins are not responsible for protective immunity induced by immunization [7, 8]. Additionally, MSP2 and MSP3 are variable among strains with strain-specific alleles encoding structurally and antigenically distinct proteins, thus these proteins are poor candidates for vaccine development [9]. Consequently, vaccine development has more recently focused on subdominant OMP antigens. Even among subdominant antigens, how best to select candidates for testing in immunization and challenge experiments is a significant question relevant to development of an effective vaccine. One of the largest outer membrane protein superfamilies in *A*. *marginale* is pfam01617, which includes MSP2 and MSP3 as well as many subdominant proteins [10]. Many of the proteins within pfam01617 are vaccine candidates because some are invariant through an infection cycle [6], are known or predicted to be surface exposed [5], are detected by immune serum from protectively immunized animals [11, 12], or are part of a complex of outer membrane proteins demonstrated to be protective [5]. While we have not focused on pfam01617 members in this study, candidate OMPs were first selected based on similar criteria, the first of which was empirical evidence of surface exposure [5, 10–15]. Additionally, we have broadened the criteria to included candidates that are either potentially functionally relevant or of which we have little *a priori* information. The later criterion reduces the bias in our choice of candidates in order to better test the hypothesis. Specifically, AM854 is a member of the complex of protective surface proteins [5]. AM854 and AM1096 are recognized by antibody in outer membrane or *A*. *centrale* vaccinates [11, 12]. The *A*. *phagocytophilum* homologs of AM854 and AM936 mediate host cell entry. Little is known about AM202, AM368 and AM1041, while AM368 and AM1041 lack homologs in other species. In this paper we report the conservation of these OMPs among isolates, their recognition by antibody from immunized and protected animals, and the result of a challenge trial following immunization with the prioritized, recombinant OMPs.

## Materials and Methods

### Sequencing of AM202, AM368, AM854, AM936, AM1041, and AM1096

A Gentra Puregene kit (Qiagen, Valencia, CA) was used to extract DNA from the blood of cattle infected with the following strains and isolates of *A*. *marginale*: Kansas 6DE [16], Dawn (Australia) [17], Colville (C51 and C52), Kansas EMФ [16], Nayarit, Mexico (N3518, N4506, and N3571) [18], Puerto Rico (PR), and Virginia (VA) [19].

Genes were amplified from genomic DNA from each strain or isolate using the primers in S1 Table and PCR Master Mix (Roche, Basel, Switzerland). Cycling conditions were as follows: initial hold at 95°C for 5 min; 27 cycles of 95°C for 30 s, 56°C for 30 s for *am936* and *am202*, 2 min for *am1041* and *am854*, and 4 min for *am368* and *am1096*, 72°C for 30 s. Next, the reactions were held at 72°C for 4 min and then at 10°C. Products from PCR were cloned for sequencing into pCR 2.1-TOPO plasmids, and TOP10 One Shot chemically competent cells following the manufacturer’s protocol for the TOPO TA cloning kit (Life Technologies, Carlsbad, CA). Plasmids were screened using the *A*. *marginale* gene-specific forward primer (S1 Table) and M13 reverse primer.

A minimum of five clones per gene was sequenced either commercially (Eurofins Operon-SimpleSeq, Huntsville, AL) or using Washington State University Genomics Core services. Clones longer than 600 base pairs required additional primers as shown in S1 Table. Vector NTI (Life Technologies, Carlsbad, CA) was used for sequence curation and analysis. Clustal Omega (EMBL-EBI) was used for multiple sequence alignments and calculation of pairwise % identity [20]. All sequences have been deposited in GenBank and have accession numbers KM821213 to KM821248, KM870519 to KM870527 and KM889598 to KM889608.

### Expression and Purification of AM202, AM854, AM936, and AM1096

Genomic DNA from *A*. *marginale* St. Maries strain was used for cloning and protein expression [10]. Primers were designed to exclude the predicted signal peptide (S2 Table). AM202 was cloned and expressed using pTrcHis TOPO vector (Life Technologies, Carlsbad, CA). AM854, AM936, and AM1096 were cloned and expressed using pBAD-D TOPO vector (Life Technologies, Carlsbad, CA), both of which have C-terminal V5 His tags that were later used for protein purification. TOP10 One Shot chemically competent cells (Life Technologies, Carlsbad, CA) were transformed with vectors containing the appropriate insert. Colonies were screened by PCR. Plasmid DNA was sequenced (Eurofins Operon-SimpleSeq, Huntsville, AL) to ensure inserts were in the correct orientation and in-frame. Protein expression was carried out following manufacturer’s instructions for both pTrcHis TOPO and pBAD-D TOPO. AM202 was induced at early log phase growth in Terrific Broth by adding IPTG to a final concentration of 10mM and incubating an additional seven hours. AM854, AM936 and AM1096 were induced at early log phase growth in Luria-Bertani broth. AM854 was induced with 2% arabinose and grown for 5 h. AM936 was induced with 20% arabinose and grown for 4 h. AM1096 was induced with 0.2% arabinose and grown for 6 h. Cultures were then pelleted at 23,500 x g for 30 minutes, supernatant was removed and pellets were stored at -80°C.

Protease inhibitor cocktail (P8849, Sigma-Aldrich, St. Louis, MO) and guanidine lysis buffer at a pH of 7.8 (6M guanidine hydrochloride, 20mM Sodium phosphate, 500 mM NaCl) were added to thawed bacterial pellets. The resulting lysate was rocked for 15 min at room temperature and lysed by repeated passage through a 15-gauge needle. The solution was sonicated (Fisher Scientific Sonic Dismembrator Ultrasonic Processor part FB705) at 200 Watts in six ten-second intervals and centrifuged at 22,000 x g for 30 min. For purification, the lysate was incubated with nickel resin (Thermo Fisher Scientific, Waltham, MA) at room temperature rocking for three hours and was subsequently added to a column per manufacturer’s directions (Life Technologies, Carlsbad, CA). To verify expression, proteins were electrophoresed on 4–20% sodium dodecyl sulfate-polyacrylamide gels (SDS-PAGE) (Bio-Rad, Hercules, CA), stained with Simple Blue (Life Technologies, Carlsbad, CA), and bands of the expected molecular weight excised from the gel and analyzed using LC-MS/MS at Washington State University proteomics core [5]. MS/MS fragments fragment ion lists were compared using a local MASCOT server to the genome of the St. Maries strain of *A*. *marginale* or NCBI. Prior to immunization, the guanidine hydrochloride was reduced to 0.7M by dilution as AM854 and AM936 precipitate in phosphate buffered saline (PBS). The final pH of the immunogen was 7.18. There was no evidence of pain or swelling at the injection sites.

### Immunoblots

To eliminate antibody directed toward remnant *E*. *coli* proteins and the C-terminal fusions tags, sera from recombinant protein vaccinates were adsorbed against lysate from *E*. *coli* expressing His-tagged pBadD LacZ. First, 700μl of serum were mixed with 300μl of lysate and allowed to incubate while rotating at 4°C for 16 h. The mixture was then centrifuged at 28,000 x g for 20 min and 700μl of the supernatant was applied to fresh 300μl of lysate. This was repeated three more times. For western blotting, 0.05μg of recombinant protein or 6x10^8^ *A*. *marginale*-infected erythrocytes per well were loaded onto 4–20% SDS-PAGE gels (Bio-Rad, Hercules, CA) and electrophoresed at 150V. After transblotting to nitrocellulose and blocking in iBlock (0.2% I-Block with 0.5% Tween-20 suspended in PBS) (Life Technologies, Carlsbad, CA) overnight at 4°C, immune and pre-immune sera were diluted with I-Block and incubated with the nitrocellulose at room temperature for 2 hours. The secondary antibody was either goat anti-bovine IgG, horseradish peroxidase (HRP) conjugate (KPL, Gaithersburg, MD) at 1: 20,000, sheep anti-bovine IgG2 HRP conjugate (Pierce-ThermoScientific, Waltham, MA) at 1: 40,000, or anti-V5 epitope HRP conjugate (Life Technologies, Carlsbad, CA) applied at 1:5,000 for one hour. The membranes were washed in I-Block and developed using ECL Western blotting Substrate (Promega, Madison, WI) following manufacturer specifications.

### Measurement of specific antibody to regions of AM854 and AM936 predicted to mediate host cell invasion

Amino acids 19–68 and 84–121 of AM854 and AM936, respectively were synthesized (NeoBioLab, Woburn, MA) and used as antigen in enzyme-linked immunosorbent assays (ELISAs). Immune sera from outer membrane and recombinant protein vaccinates were tested. Immulon-II 96 well plates were coated with 1μg of peptide per well with a carbonate coating buffer (15mM Na2CO3, 35mM NaHCO3 pH9.6) overnight at 4°C, washed with PBS containing 0.05% (vol/vol) Tween20 and then blocked with PBS containing 0.05% (vol/vol) Tween20 with 5% bovine serum albumin fraction V (Sigma-Aldrich, St. Louis, MO) for 2 hours. Each well received 50ul of diluted serum and samples were run in triplicate. Antibody binding was detected using 50μl of 1:500 dilution of anti-bovine IgG labeled with bovine-horseradish peroxidase (KPL, Gaithersburg, MD) and developed with SureBlue TMB Microwell Peroxidase Substrate (KPL, Gaithersburg, MD). The optical density at 450nm was determined. To determine the end point titers, sera were diluted starting at 1:10 in blocking buffer. Dilutions were doubled until a signal was no longer detected. Positive binding was statistically defined as exceeding the mean plus three standard deviations of the OD450 of pre-immune serum from the same animal for the same peptide. Titers were reported as the reciprocal of the last dilution in which antibody binding was detected. One microgram per well of full-length recombinant AM854, described above, was used as a positive control for the AM854, amino acids 19–68 ELISAs. For the recombinant protein vaccinates, adsorbed serum at a dilution of 1:80 was used. For the outer membrane vaccinates a serum dilution of 1:80 was used for all animals except 071 (positive at 1:10) and 105 (negative) in which serum dilutions of 1:10, 1:80, and 1:160 were tested.

### Isolation of *A*. *marginale* outer membranes

Outer membranes were extracted from *A*. *marginale*-infected red blood cells (St. Maries isolate) obtained from an acutely infected calf using a previously described protocol [4, 5, 21].

### Animals

The cattle used in this study were treated in strict accordance to guidelines set by Washington State University Animal Care and Use Committee. The protocol was approved by the Washington State University Animal Care and Use Committee (ASAF #3686). All calves were age-matched and confirmed to be negative for *A*. *marginale* based on cELISA [22]. Alleles for the *DRB3* gene were determined by direct sequencing of PCR product, using published primers [23]. DNA was extracted from whole blood (Qiagen, Valencia, CA) as described above. The *DRB3* alleles were amplified using PCR [23, 24] and the PCR product was sequenced (Eurofins Operon-SimpleSeq, Huntsville, AL). Allele assignment was done using Assign ATF 1.5 (Conexio Genomics, Fremantle, Australia). Animals were divided into three groups of five animals each to be immunized with recombinant protein, outer membrane protein, or adjuvant only. All fifteen steers were half-matched and had DRB3 allele *0101. Each group was assigned one animal with the following haplotypes: *0101/*0101, *0101/*1501, *0101/*1101,* 0101/*1001, and *0101/*1401. The exception being that two animals in the group that received only adjuvant were *0101/*1201 and *0101/*0102 instead of *0101/*1401 and *0101/*0101, respectively.

### Immunization

The first group was immunized with 30μg each of AM936 and AM854 suspended in 1mg/ml saponin. The second group, which served as a positive control for protective immunity, received 60μg of outer membranes suspended in 1mg/ml saponin. The third group, which served as a negative control, received 1mg/ml of saponin. Animals were immunized subcutaneously 4 times at three week intervals.

### Challenge

Calf 43811 was inoculated with 1.8ml of St. Maries-strain stabilate from C5321BL suspended in 4ml of serum. When the percent of infected erythrocytes reached 3.3% in calf 43811, *Dermacentor andersoni* (Reynold’s Creek colony) ticks were applied for an acquisition feed of seven days during which the percent of infected erythrocytes in the donor calf reached 4%. After feeding, ticks were incubated for seven days at 26°C to allow for digestion of the blood meal. Thirty days following the last immunization, ten acquisition-fed ticks were applied to each immunized animal and allowed to feed for seven days. Day one post challenge was counted as the day the ticks were applied to the animals. Blood was collected from the animals every day for 40 days starting seven days after the ticks were removed. The packed cell volume (PCV) and the percent of infected erythrocytes was determined daily by counting bacterial inclusion bodies in Giemsa-stained blood smears using light microscopy.

After feeding, the ticks used for challenge were dissected, and DNA was extracted from salivary glands using a Gentra Puregene kit (Qiagen, Valencia, CA). The *A*. *marginale* infection level in the ticks was determined using digital drop PCR (Bio-Rad, Hercules, CA). Primers used were 5’CCGAAGTTGTAAGTGAGGG3’F and 5’CTTATCGGCATGGTCGCC3’R at a 10μM concentration. The probe used was 5’/-FAM/CCTCCGCGTCTTTCAACAATTTGGTT/-TAMSp/3’ at a 2.5μM concentration. Digital drop PCR supermix (Bio-Rad, Hercules, CA) was used at 1x concentration. The cycling parameters were as follows: 95°C for 10 min, 94°C for 30 s, and 60°C for one min each for 40 cycles followed by a 10 min hold at 98°C. QuantaSoft software was used to analyze the data from the reader.

### Statistics

Statistical significance in the differences between titers as measured using western blotting and ELISAs was determined using the Kruskal-Wallis test based on data ranks blocked by treatment and JMP version.10.0.0 (SAS Institute Inc., Cary, NC). Statistically significant differences between individual groups were then determined using the Wilcoxon test for each pair. For the immunization and challenge experiment, the daily percentage of infected erythrocytes and packed cell volume for each animal for days 30 to 43 post challenge were included in the calculation of the mean percent infected erythrocytes and mean PCV for each group. A repeated measures analysis of variance (ANOVA) with a heterogeneous autoregressive covariance structure was used to determine if differences in percent infected erythrocytes and PCV were statistically significant when comparing treatment groups. The statistical model included treatment, days post challenge, treatment by days post challenge (treatment*days post challenge), and the random effect of animal nested within treatment (animal [treatment]). Post hoc testing was done using Fisher’s least significant difference (LSD). These analyses were performed using SAS software version 9.4 (SAS institute Inc., Cary, NC).

An additional ANOVA was done to identify the individual days that were statistically significantly different. This was done with two caveats. First, the effect of treatment by days post challenge was not statistically significant thus post hoc testing to determine pairwise differences among days is not appropriate. Second, this analysis fails to account for the fact that the daily measurements from the same animal are not independent one from another. These analyses were performed using SAS software version 9.4 (SAS institute Inc., Cary, NC).

## Results

### Conservation of OMPs among diverse isolates

The six proteins AM202, AM368, AM854, AM936, AM1041 and AM1096 were initially selected based on empirical evidence of surface exposure and, for AM854 and AM936, homology to *A*. *phagocytophilum* OMPs, OmpA and Asp14, shown to mediate entry into host cells *in vitro* [13, 14]. The level of conservation of these proteins was evaluated using six well-characterized *A*. *marginale sensu stricto* strains, five from the U.S. (Florida, St. Maries, Kansas 6DE, Kansas EMΦ, Virginia), one from Puerto Rico, and one from Australia (Dawn), and five more recent isolates two of which are from the U.S. (Colville, WA; C51, C52) and three from Mexico (Nayarit; N3571, N3518, N4506). In addition, sequences were compared to *A*. *marginale* ss. *centrale*, a less virulent subspecies used as a cross-protective live vaccine for over 100 years [1, 25]. The complete genome sequences of the St. Maries (CP000030) and Florida (CP001079) strains and *A*. *marginale* ss. *centrale* (NC_013532.1) were available in GenBank. The targeted OMP sequences for the remaining strains and isolates were determined by PCR amplification, cloning, and sequencing. The amino acid alignments and percent pairwise identities for each protein are shown in the supplementary data (S3–S8 Tables and S1–S6 Figs).

Among the *sensu stricto* strains and isolates there is 100% amino acid identity for AM202 and AM936 (Table 1). As expected and previously reported for strains from North America, AM854 is highly conserved [12, 26]. In this study, the identity for AM854 is also 100% except for EMΦ in which a single, non-conservative amino acid substitution results in 99.6% identity with all other isolates and strains. Similarly in AM1096 a single non-conservative amino acid substitution in three different positions involving four strains or isolates results in the observed variation and a minimum identity of 99.6% (Table 1, S3–S8 Tables, S6 Fig). For AM1041 and AM368, overall more strains or isolates have more single amino acid substitutions resulting in fewer strains and isolates having 100% identity. AM368 is the only protein in which none of the strains or isolates share 100% identity, though the minimum identity between any two sequences is high at 97.8%.

**Table 1:** Pairwise percent identity scores for each candidate protein.

	*A*. *marginale*			*A*. *marginale* ss. *centrale*
Protein^a^	Average^b^	Minimum^c^	Maximum^d^	Average^e^
AM202	100.0%	100.0%	100.0%	99.0%
AM368	98.6%	97.8%	99.9%	38.2%
AM854	99.9%	99.6%	100.0%	78.8%
AM936	100.0%	100.0%	100.0%	100%
AM1041	98.6%	97.1%	100.0%	98.1%
AM1096	99.9%	99.6%	100.0%	87.7%

^a.^ The St. Maries strain locus tag for each candidate protein.

^b.^ Average of the pairwise identity scores for each protein among all *sensu stricto* strains and isolates.

^c.^ Minimum of the pairwise identity scores for each protein among all *sensu stricto* strains and isolates.

^d.^ Maximum of the pairwise identity scores for each protein among all *sensu stricto* strains and isolates.

^e.^ Average of the pairwise identity scores for each protein when comparing all *sensu stricto* sequences to the *A*. *marginale* ss. *centrale* ortholog.

The percent identities between the *A*. *marginale* proteins and the *A*. *marginale* ss. *centrale* orthologs are much more variable, as would be expected. The highest conservation is between AM936 and the *A*. *marginale* ss. *centrale* ortholog, ACIS_00403 which share 100% identity (Table 1). The identity between AM202 and its ortholog, ACIS_01081, is also high with two amino acid substitutions resulting in 99.0% identity. AM1041 and the *A*. *marginale ss*. *centrale* ortholog ACIS_00314 are highly conserved with 98.1% identity. There is approximately 87.7% identity in AM1096 among all *sensu stricto* strains and isolates and the *A*. *marginale* ss. *centrale* ortholog ACIS_00268 with several regions of greater than 25 amino acids throughout the protein that have 100% identity. Unlike AM854 in the *sensu stricto* strains and isolates, the conservation between AM854 and the *A*. *marginale* ss. *centrale* ortholog ACIS_00486 is relative low at 78.8%. There is complete identity from amino acids 37–72 and 80–128 and 130–152, representing approximately 45% of the St. Maries protein. Despite the overall low amino acid identity of 38% with AM368, ACIS_00938 is the ortholog in *A*. *marginale* ss. *centrale* because based on BLAST alignments this gene has the lowest Expect value of any open reading frame in the genome (3e-69), the locus is conserved and both AM368 and ACIS_00938 are downstream of *uvrD* in their respective genomes.

### OMP recognition by antibody from protected vaccinates

Based on the conservation among *sensu stricto* strains and isolates and with the *A*. *marginale* ss. *centrale* vaccine strain, OMPs AM202, AM854, AM936, and AM1096 were used for immunological analysis. Sera from animals that were immunized with *A*. *marginale* outer membrane proteins, linked surface protein complexes, or unlinked surface protein complexes [4, 5, 21] were used to determine if antibody to these candidate OMPs was produced in response to vaccination. The sera were collected two weeks following the last immunization from vaccinates which were later shown to be protected. These immunizations and results have previously been reported [5, 21]. AM202, AM854, AM936, and AM1096 were individually cloned, expressed in *E*. *coli*, and purified as recombinant proteins. Only single bands on Coomassie-stained gels were observed following purification and separation using SDS-PAGE. Mass spectrometry analysis of the excised bands identified the expected protein. Using western blot analysis with individual recombinant proteins, AM854 was the most widely recognized with 14 of 15 (93%) vaccinates having anti-AM854 IgG antibodies, with a mean titer of 2,673 and a median titer of 3,000 (Table 2). AM936 was recognized by 9 of 15 (60%) vaccinates having anti-AM936 antibody, with a mean titer of 5407 and a median of 1,000 (Table 2). In contrast, less than half of vaccinates recognized AM202 (5/15, 33%) and AM1096 (4/15, 27%). Those that did respond had low titers, with means of 887 and 127 for AM202 and AM1096, respectively, and both with median titers of <100 (Table 2). The mean titers for both AM854 and AM936 were statistically significantly greater (p = 0.02) as compared to titers to AM202 and AM1096. There was no statistically significant difference between mean titers to AM854 and AM936 (p = 0.93). Consequently both AM854 and AM936 were selected for testing by immunization and challenge.

**Table 2:** IgG titers to recombinant, candidate proteins in protectively immunized animals.

Immunogen	Animal No.	AM202	AM854	AM1096	AM936
Outer membrane proteins^a^	5953	1000	3000	1000	10000
	5966	10000	10000	300	3000
	5975	1000	10000	300	30000
	5978	300	3000	<100	3000
	5982	1000	3000	300	30000
Linked^b^	5933	<100	3000	<100	<100
	5946	<100	300	<100	1000
	5952	<100	3000	<100	100
	5961	<100	3000	<100	1000
	5972	<100	300	<100	3000
Unlinked^c^	35095	<100	300	<100	<100
	35142	<100	<100	<100	<100
	35149	<100	1000	<100	<100
	35150	<100	100	<100	<100
	35164	<100	100	<100	<100

^a.^ Titers in outer membrane protein vaccinates shown to protected from challenge.

^b.^ Titers in linked surface protein complex vaccinates shown to be protected from challenge.

^c.^ Titers in unlinked surface protein complex vaccinates shown to be protected from challenge.

### Response to immunization

Holstein calves were immunized with recombinant AM854 and AM936. As a positive control, calves were immunized with purified outer membranes [4]; as a negative control, calves were immunized with adjuvant alone. All calves (n = 5/group) were seronegative prior to immunization, age-matched, and half- or fully-matched at the DRβ3 allele. Following the final booster immunization, the response to the respective immunogen was confirmed.

**Fig 1:**
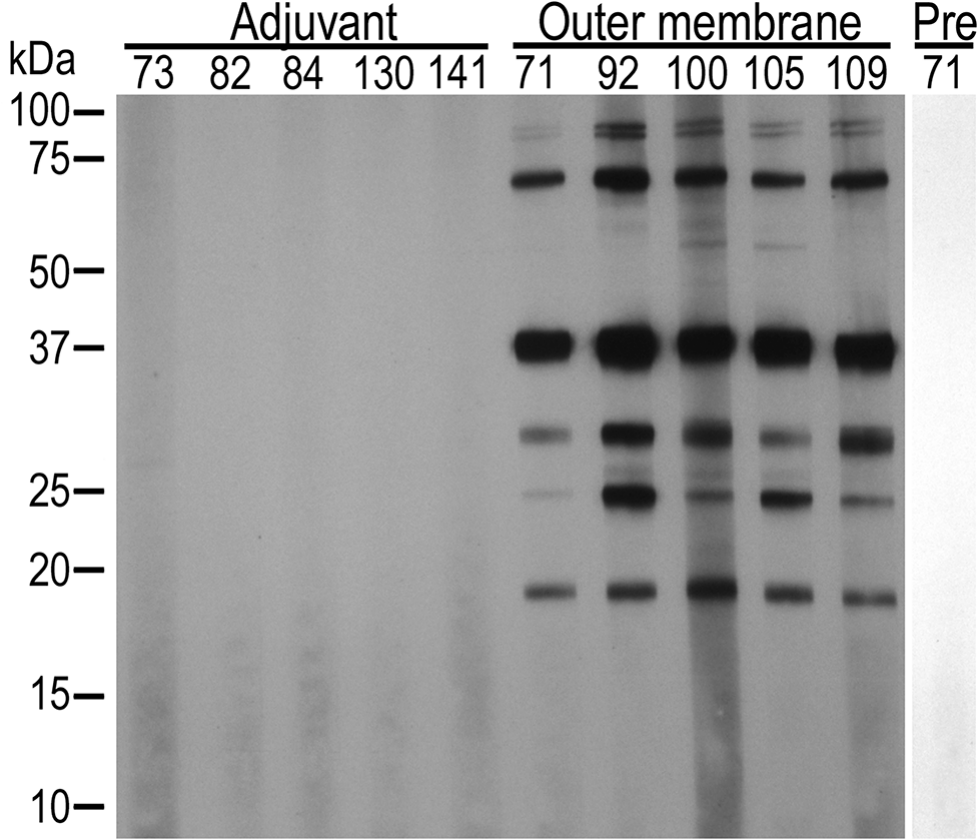
Western blot demonstrating a specific antibody response to native *A*. *marginale* proteins in vaccinates. *A*. *marginale*-infected erythrocytes loaded at 1x 10^8^ infected red blood cells per well are used as antigen. Outer membrane vaccinates (Outer membrane) have antibody directed against multiple antigens, as expected. No anti-*A*. *marginale* antibodies are detected in the group that received adjuvant alone (Adjuvant) or prior to immunization (Pre), shown for animal 71. All western blots were done with a serum dilution of 1:100. Animal numbers are listed across the top.

**Fig 2:**
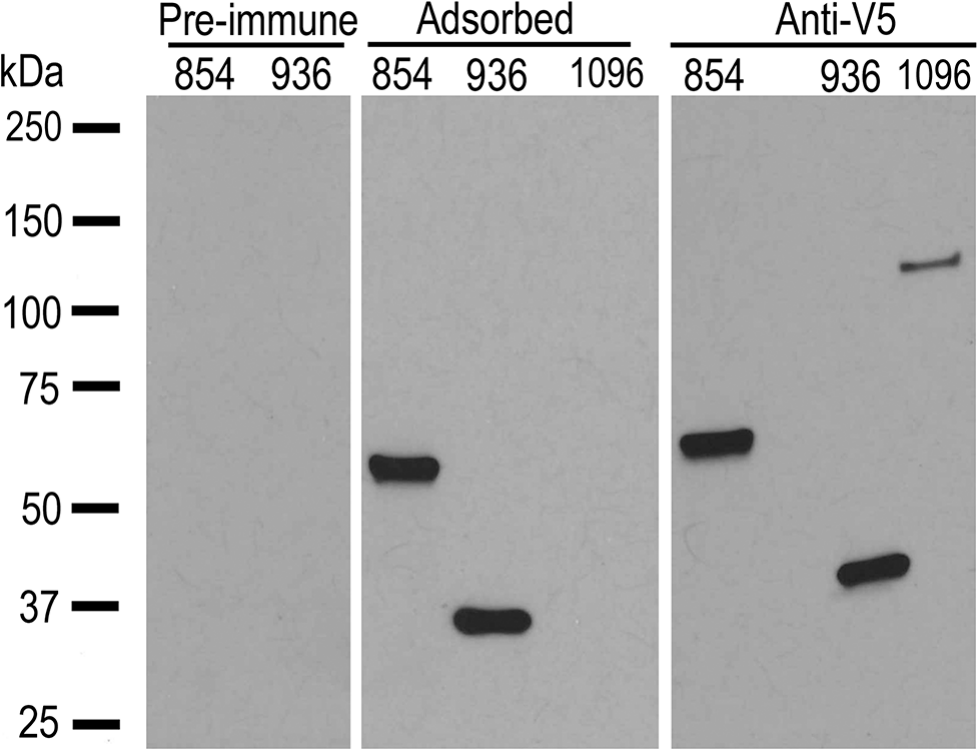
Western blot demonstrating specific response to recombinant AM854 and AM936 in AM854/AM936 vaccinates. Serum was adsorbed against protein lysates from the *E*. *coli* expression vector and His-tagged LacZ prior to western blotting. Recombinant AM854 (54kDa), AM936 (36kDa) and as a negative control, AM1096 (100kDa), were used as antigen. There is no antibody response to any of the proteins prior to immunization (Pre-immune). After immunization and adsorption (Adsorbed), anti-AM854 and anti-AM936 antibody, but no antibody directed toward AM1096 or the fusion tags was detected. One representative animal, 146, is shown. A serum dilution of 1:100 was used in all western blots. The anti-V5 antibody at a dilution of 1:500 demonstrates the presence of recombinant protein in each well.

**Fig 3:**
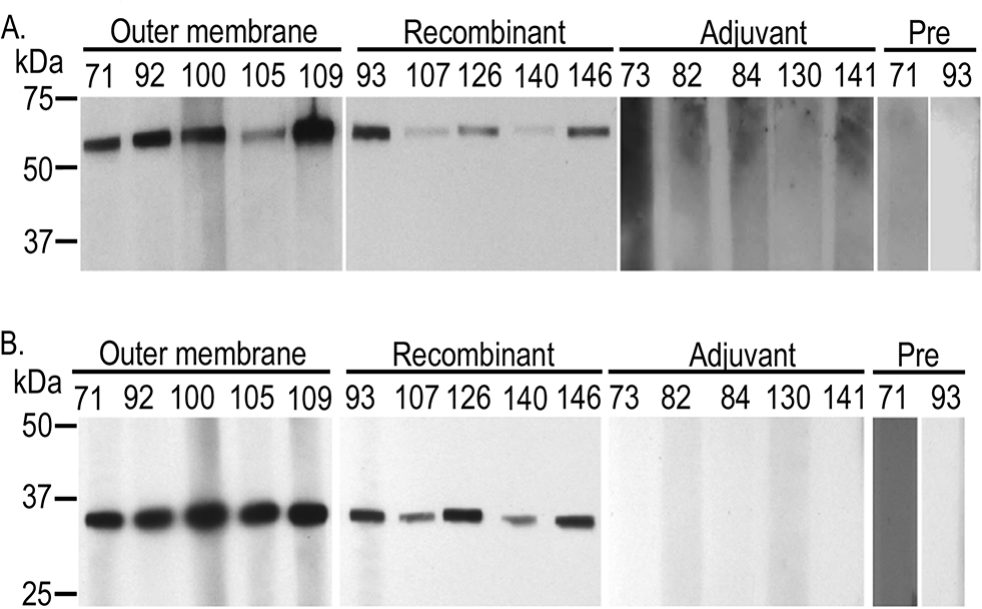
Western blots demonstrating IgG response to recombinant proteins in vaccinates. Animals immunized with purified outer membranes (Outer membrane) or recombinant proteins (Recombinant) had an antibody response to (A) recombinant AM854 at 54kD and (B) recombinant AM936 at 36kD. There was no response to these proteins in animals that received only adjuvant (Adjuvant). Similarly, there was no response prior to immunization (Pre). Results for all animals prior to immunization were similar, thus one representative animal from each group is shown. All western blots were done using a serum dilution of 1:100.

The outer membrane but not the adjuvant only vaccinates developed antibody to native whole *A*. *marginale* (Fig 1). The pattern of response as determined by western blotting was similar to that previously reported [4, 5, 21]. Serum from AM854/AM936 vaccinates was first adsorbed against *E*. *coli* proteins including a recombinantly expressed His-tagged LacZ to ensure removal of antibodies directed against contaminating *E*. *coli* proteins and the C-terminal fusion tags (Fig 2). After adsorption, no antibody was detected against His-tagged AM1096, demonstrating successful removal of anti-*E*. *coli* and anti-His-tag antibodies. All AM854/AM936 vaccinates had antibodies directed against both recombinant OMPs (Fig 3). There was no response to either protein prior to immunization. Adjuvant only vaccinates had no response to recombinant proteins (Fig 3).

Immunization with purified, native outer membranes resulted in an antibody response in all vaccinates to recombinant AM854 and AM936 (Fig 3), confirming these two proteins were present in the protection-inducing outer membrane immunogen. To determine if the antibody response was similar in outer membrane and recombinant AM854/AM936 vaccinates, total IgG and IgG2 titers were measured using recombinant proteins as antigen and sera adsorbed against *E*. *coli* lysates (Table 3). IgG2 was specifically measured because IgG2 titers have been reported to correlate with protective immunity to *A*. *marginale* challenge [3]. In the outer membrane vaccinates, the mean IgG titer to AM854 was 540 with a median of 300; the mean IgG2 titer was 160 with a median of 100. These were not significantly different from the titers to AM854 in the AM854/AM936 vaccinates which had mean IgG and IgG2 titers of 580 and 220, respectively, with medians of 300 in both cases.

**Table 3:** IgG and IgG2 directed against recombinant AM854 and AM936 following immunization with recombinant proteins, purified outer membranes or adjuvant alone.

			AM854		AM936	
Immunogen	Animal No.	*DRB3* haplotype	IgG	IgG2	IgG	IgG2
Recombinant Proteins	93	*0101/*0101	1000	300	3000	1000
	107	*0101/*1401	300	300	1000	1000
	126	*0101/*1101	300	100	3000	1000
	140	*0101/*1001	300	100	1000	1000
	146	*0101/*1501	1000	300	3000	1000
Outer membrane proteins	71	*0101/*0101	300	100	3000	3000
	92	*0101/*1501	1000	300	3000	1000
	100	*0101/*1001	300	100	3000	1000
	105	*0101/*1401	100	<100	1000	1000
	109	*0101/*1101	1000	300	30000	30000
Adjuvant only	73	*0101/*1501	<100	ND	<100	ND
	82	*0101/*1002	<100	ND	<100	ND
	84	*0101/*1201	<100	ND	<100	ND
	130	*0101/*1101	<100	ND	<100	ND
	141	*0101/*1001	<100	ND	<100	ND

Overall the titers to AM936 were higher than those to AM854, but similar in both the outer membrane and recombinant AM854/AM936 vaccinates. The outer membrane vaccinates had mean IgG and IgG2 anti-AM936 titers of 8,000 and 7,200, respectively, with medians of 3,000 and 1,000. The recombinant vaccinates had mean anti-AM936 titers of 2,200 for IgG and 1,000 for IgG2, with medians of 3,000 and 1,000, respectively (Table 3). In summary, the IgG and IgG2 titers to recombinant AM854 and AM936 were not significantly different between the outer membrane and recombinant OMP vaccinates.

**Fig 4:**
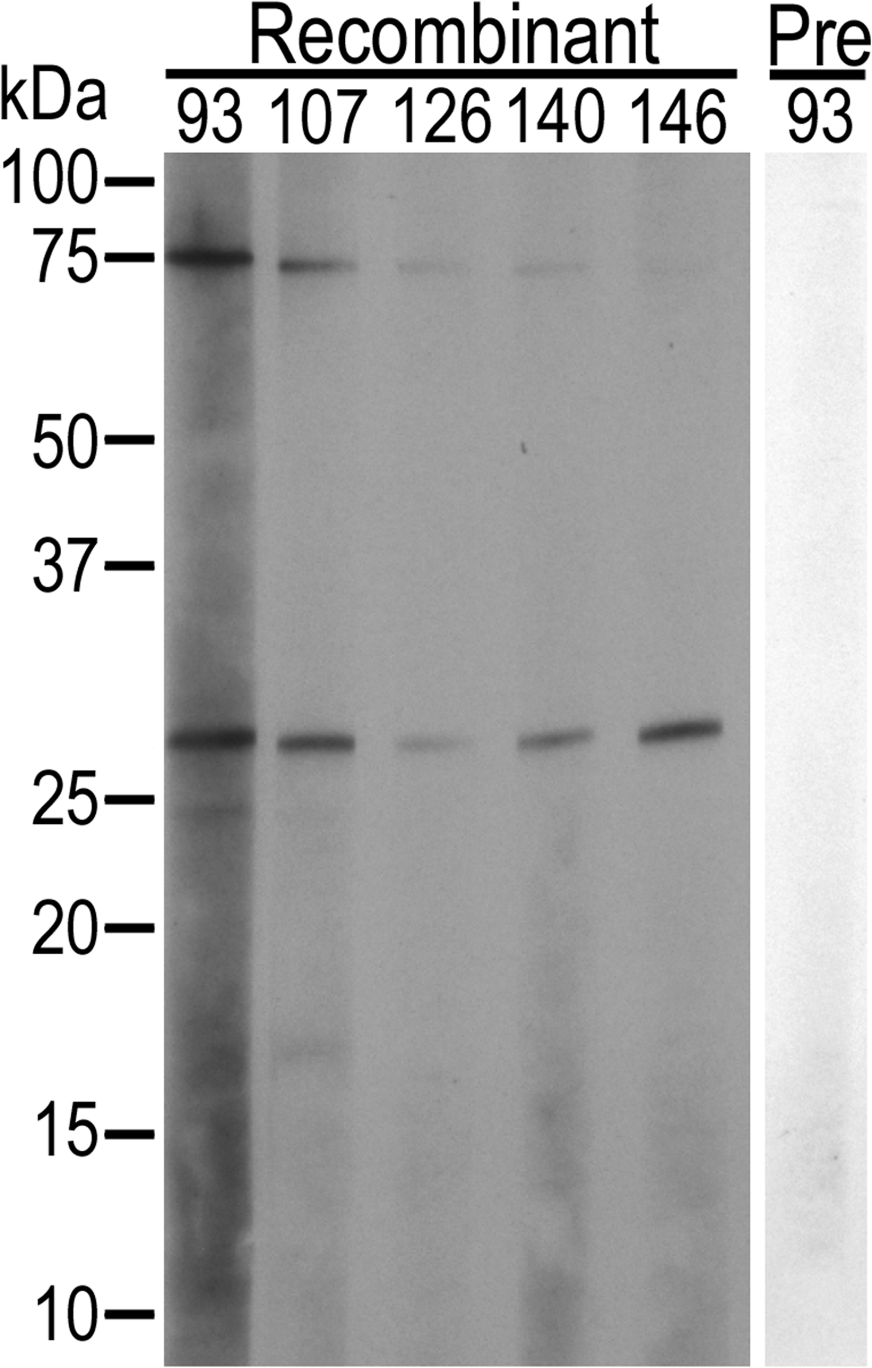
Western blot demonstrating a specific antibody response to native *A*. *marginale* in AM854/AM936 vaccinates. *A*. *marginale*-infected erythrocytes loaded at 1x 10^8^ infected red blood cells per well are used as antigen. Recombinant AM854/AM936 vaccinates (Recombinant) have antibody directed toward an approximately 26kDa antigen, confirmed to be AM854 by mass spectrometry. A band at the predicted size for AM936, approximately 14kDa, is not identified. The band at 75kDa was identified as a bovine ATP synthase by mass spectrometry. No anti-*A*. *marginale* antibodies are detected in the animals prior to immunization (Pre), shown for animal 93. All western blots were done with a serum dilution of 1:100. Animal numbers are listed across the top.

To determine if immunization with recombinant AM854/AM936 generated an antibody response to native AM854 and AM936, western blots were done using whole *A*. *marginale* derived from infected bovine erythrocytes (Fig 4). Only native AM854 was bound by antibody from all recombinant AM854/AM936 vaccinates. The identity of AM854 was confirmed by mass spectrometry (MASCOT score 252). Binding to native AM936 was not detected on the western blots using a minimum serum dilution of 1:100. Native AM936 was not detected by mass spectrometry in a faint band of the predicted size (approximately 13kD) from a Coomasie-stained gel of whole *A*. *marginale* suggesting this protein is below detectable limits by both western blotting and mass spectrometry.

In *A*. *phagocytophilum*, the invasion domain of OmpA, the homolog of AM854, and Asp14, the homolog of AM936, include aa59-74 of OmpA, and aa113-124 of Asp14 [27]. In order to determine if the antibody response directed against the predicted invasion domains of our test proteins could account for the difference in protection observed in the outer membrane and AM854/AM936 vaccinates, end-point IgG titers as determined by ELISAs were compared between the two groups of vaccinates. However, invasion domains have not been identified in AM854 and AM936. Consequently, the length of the tested peptides was extended to include the predicted extracellular domain of OmpA (aa19-74) and a longer conserved region of Asp14 (aa101-124) [13, 14]. Alignments were then done to identify the corresponding amino acids in St. Maries AM854 (aa19-68) and AM936 (aa84-121) (S7 and S8 Figs).

In the case of AM854 aa19-68, of the AM854/936 vaccinates, only one animal had a detectable titer of 80 (Table 4), though all animals had a positive response to full-length recombinant AM854 as detected by ELISA. No response was detected in the outer membrane vaccinates, though all had a detectable antibody to full-length recombinant AM854 with the exception of animal 105. This animal also had low total IgG titers of 100 to recombinant AM854 as determined by western blotting. In the case of AM936 aa84-121, the outer membrane vaccinates had a mean IgG titer of 5360 and a median titer of 1600. The AM854/AM936 vaccinates had a mean and median titer of 3200 and 1600, respectively. Though the outer membrane vaccinates had higher titers, these differences were not statistically significant.

**Table 4:** IgG directed against predicted host cell binding domains of AM854 and AM936 following immunization with recombinant proteins or purified outer membranes.

		AM854 aa19-68	AM936 aa84-121
Immunogen	Animal No.	IgG	IgG
Recombinant Proteins	93	<10	1600
	107	<10	6400
	126	<10	800
	140	<10	800
	146	80	6400
Outer membrane proteins	71	<10	800
	92	<10	6400
	100	<10	400
	105	<10	6400
	109	<10	12800

### Response to challenge

For challenge, ten *A*. *marginale* exposed ticks were allowed to feed on the immunized animals for 7 days. Following this transmission feed, infection in all ticks was confirmed and quantified using real time PCR. The infection level in the salivary glands was similar among all groups of ticks. Specifically, the mean infection level in the ticks that transmission fed on the outer membrane and AM854/AM936 vaccinates was (mean +/- standard deviation) 4.26 x 10^4(+/-0.45)^ and 4.73 x 10^4 (+/- 0.720)^, respectively. The infection level was 6.06 x 10^4 (+/- 0.49)^ in the ticks that fed on the animals that received only adjuvant.

**Fig 5:**
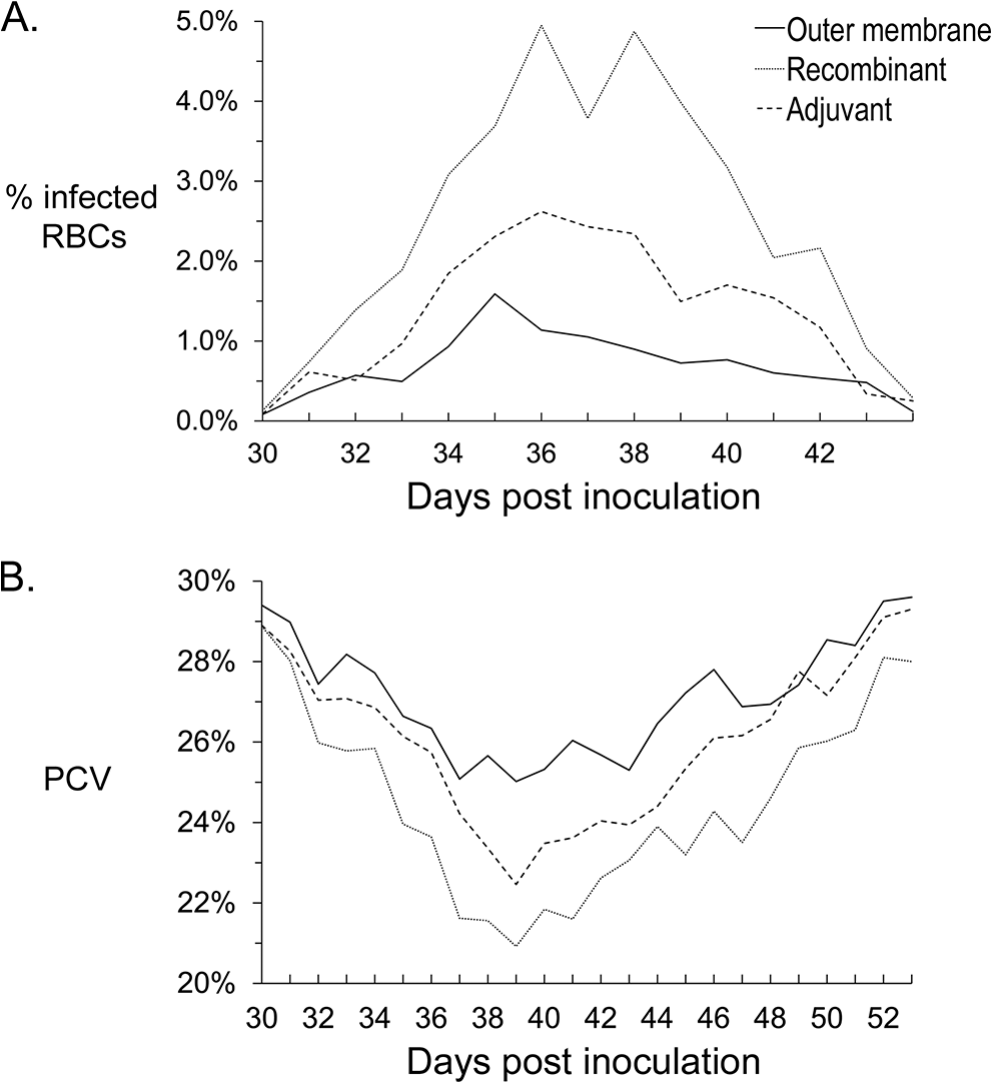
Percent infected erythrocytes and PCV in animals over time during challenge. The means of the percent infected erythrocytes (A) or PCV (B) for each group of animals are plotted by days post challenge. The AM854/AM936 vaccinates developed a statistically significantly higher percent of infected erythrocytes than the outer membrane vaccinates (p = 0.018). The differences in PCV were not statistically significant.

All 15 animals, regardless of vaccination group, became infected with *A*. *marginale* as determined by microscopic identification of cell-associated bacteremia (Fig 5A and S9 Fig). The levels and duration of bacteremia were compared among groups for the period in which the percentage of infected erythrocytes was ≥1% in any animal, which corresponded to days 30 to 43 post-challenge. Surprisingly, the AM854/AM936 vaccinates developed the highest bacteremia, with a mean (+/- SEM) percentage of infected erythrocytes of 2.64% (±0.404%). The mean of the maximum percent infected erythrocytes for this group was 6.50%. These levels were statistically significantly greater than those in either the adjuvant-only vaccinates or the native outer membrane vaccinates (p = 0.018). The native outer membrane vaccinates developed a mean (±SEM) percentage of infected erythrocytes of 0.73% (±0.404%), with a mean of the maximum percent infected erythrocytes of 1.83%. These levels were not statistically significantly different as compared to the adjuvant-only vaccinates, which had mean levels of 1.44% (±0.404%) infected erythrocytes, and a mean of the maximum percent infected erythrocytes of 3.53%.

The level and duration of anemia as measured by PCV was compared between groups for the same period (days 30 to 43 post challenge) (Fig 5B and S10 Fig), and the mean (± SEM) PCV was calculated for each group. Consistent with the bacteremia levels, the recombinant AM854/AM936 vaccinates developed the most severe anemia with a mean PCV of 24% (±1.30%), which corresponds to a 22% drop in PCV. In the native outer membrane vaccinates, the mean PCV was 27% (±1.30%), which represents an18% drop in PCV. The group that received only adjuvant had a mean PCV of 25% (±1.30%), a 20% overall drop in PCV. The differences among the groups was not statistically significant (p = 0.375).

## Discussion

The identification of protective antigens remains a major limitation in the development of vaccines particularly those targeting pathogens that cause persistent infection. In the experiments reported here we used a combination of predicted gene function and reverse genetics coupled with antibody response to candidates in protectively immunized animals to prioritize vaccine candidates. The functional predictions led to the identification of AM854 and AM936. Interestingly, these two proteins were also the most highly conserved in the *sensu stricto* strains and isolates and produced the most robust antibody response in immunized animals. These two proteins, which are predicted to mediate host cell entry, are also highly conserved among the *Anaplasmataceae* [13, 14].

The antibody response to immunization was as expected with the exception that native AM936 was not detected by western blotting using *A*. *marginale* infected erythrocytes as antigen and immune serum from AM854/AM936 vaccinates. However, the outer membrane vaccinates produced antibody that binds recombinant AM936, demonstrating this protein is present, at least in minimally detectable levels, in outer membrane preparations and is immunogenic. In comparison, the *A*. *phagocytophilum* homolog, Asp14, can be detected using western blots in *A*. *phagocytophlium* grown in HL-60 cells and immune serum raised against recombinant proteins [13]. The reason for the discrepancy in the ability to detect these two related proteins is unknown. However, immunization with recombinant AM936 likely results in production of a repertoire of antibodies that are a subset of those that would result from immunization with native proteins. It is unlikely that this antibody repertoire would completely fail to bind native AM936 when in a SDS-PAGE western blot. Thus, the most likely explanation for the lack of detection of native AM936 is that this small protein (13 kDa) is present at levels below that of detection of western blotting and mass spectrometry. Additionally, AM936 has not been detected in three separate proteomic analysis that included either surface proteins or whole *A*. *marginale* protein extracts derived from infected erythrocytes and tick cell culture [5, 11, 28].

When tested in an immunization and challenge trial, recombinant AM854 and AM936 failed to induce protective immunity. In vaccine development studies, reporting negative results is essential to avoid others duplicating ineffective protocols and help in understand why predicted effective approaches were unsuccessful [29, 30]. There are several possible explanations for the lack of protective immunity in this study. First it is possible that the predicted binding domains of AM854 and AM936 were poorly recognized in the AM854/AM936 vaccinates as compared to the outer membrane vaccinates thus potentially accounting for the differences in protection. However, this possibility was tested and found not to be the case as the antibody response to each of these domains was similar in these two groups of vaccinates, though the response to the AM854 domain was nearly absent.

Second, though the titers to both AM854 and AM936 in the outer membrane and AM854/AM936 vaccinates were similar, measurement of the antibody response using immunoblots and ELlSAs may not have captured critical epitopes, especially if these epitopes are highly conformational or require tertiary or quaternary structure. Thus, important differences in the antibody response between the outer membrane and AM854/AM936 vaccinates may not have been detected. On a similar note, the functional domains of AM854 and AM936 have not been empirically identified and it is unknown if the antibodies directed against these proteins target functionally essential regions.

Third, the total IgG and IgG2 titers to both AM854 and AM936 were low as compared to typical titers in outer membrane vaccinates, which tend to be ≥10,000 [4, 5, 21]. It is possible that the lack of protection was due to insufficient titers. However, in the most recent studies, titers do not correlate with protection from challenge [4,5]. For example, vaccinates with total IgG titers of ≥10,000 were similarly protected as animals with titers of 300. Animals with IgG2 titers of ≥10,000 were similarly protected as those with titers that varied from <100 to 1,000.

Another possibility is that AM854 and AM936 may be masked in the live bacterium and thus not available to the protective immune response. Alternatively or additionally, multiple surface proteins are likely to mediate cell entry, thus targeting just two of these proteins may be insufficient to prevent cell entry [13, 14, 31].

Not only did the recombinant proteins fail to induce protective immunity, the AM854/AM936 vaccinates developed a higher infection level than the animals that received only adjuvant. Several immunization and challenge experiments have been done using both *A*. *marginale* affinity purified and recombinant *A*. *marginale* surface proteins. While induction of protective immunity has varied among trials based on immunogen vaccine composition and vaccination regimen, an increased level of infection has not been observed [32]. Induction of tolerance toward otherwise protective epitopes could result in increased infection levels. In this case, the immunization schedule, including dose, route, adjuvant used and frequency of immunization, was similar to previous studies using recombinant proteins [33–35]. Additionally induction of tolerance generally requires large amounts (6mg or more) of antigen [36, 37]. Therefore, tolerance appears to be an unlikely explanation. Alternatively, the increase in bacterial levels may be due to either the production of blocking antibodies in response to immunization with the recombinant proteins or antibody-dependent enhancement of disease. In the former, blocking antibodies combine with an antigen without effecting neutralization or clearance, but block other antibodies from binding to a protective epitope. For example, binding of anti-AM936 or anti-AM854 antibody to their respective epitopes may have resulted in masking or blocking access to protective epitopes. Such a phenomenon has been reported in *Plasmodium falciparum* where IgM binding to infected erythrocytes blocks binding of specific monoclonal antibodies to protective epitopes but does not impair function of the bound protein [38]. It has also been postulated that anti-tumor antibody can coat tumor cells, thus protecting those cells from cytotoxic T lymphocytes [39].

Antibody-dependent enhancement of disease is a well-described phenomenon that occurs with many viral infections. Disease enhancement occurs via several mechanisms and most commonly in viruses that infect macrophages, but has also been described in bacterial infections [40, 41]. For example, modification of IgA1 antibody bound to a capsular polysaccharide of *Streptococcus pneumoniae* by a bacterial protease resulted in increased adherence of the bacterium to the host cell [42]. Certain monoclonal antibodies directed against Protective Antigen, a component of the *Bacillus anthracis* exotoxin, enhance the lethality of this toxin [43]. Such mechanisms have not been described for obligate intracellular bacteria, however, it is possible that antibody binding to specific surface proteins enhances entry of the bacteria into the host cell.

In this study, the outer membrane vaccinates were statistically significantly protected as compared to the AM854/AM936 vaccinates, however the outer membrane vaccinates were not statistically significantly protected from bacteremia as compared to the adjuvant only vaccinates. The reason for this failure of protection is unknown. One possibility is the limitation in the number of animals per group and the variation among animals in the development of clinical anaplasmosis led to insufficient power to detect statistically significant differences. Alternatively, in the majority of immunization experiments, challenge has been done using intravenous inoculation. In this study and a study by Saleh et al, challenge was done using infected ticks. Because tick salivary secretions are immunomodulatory, it is possible that tick feeding potentiates infection such that the level of protection achieved with any immunogen is diminished [33, 44, 45].

The most successful vaccines target highly conserved epitopes that are often on proteins required for host cell entry [46, 47]. As such, the utility of AM854 and AM936 as effective vaccine antigens cannot be dismissed based on these experiments. Immunization experiments particularly with recombinant proteins are complex because of the many variables that can influence the outcome. These variables include the adjuvant, particulate size of the antigen, a potential lack of or inappropriate folding of the antigens, absence of appropriate post-translational processing, such as glycosylation and addition of lipid moieties, route of immunization and route and dose of challenge [21, 48]. Apart from particular viral pathogens in which *in vitro* neutralization assays serve to predict relevance of an immune response, strong correlates of immunity are generally lacking for many types of pathogens, and particularly for vector borne pathogens that establish persistent in the mammalian host. Additionally, the overall lack of knowledge of the proteins and dynamic protein-protein interactions required for host cell entry and the induction of protective immunity limit our understanding of how to block rather than potentiate this process. Overall the findings reported here in conjunction with examples of successful vaccines, point to knowledge gaps that when filled, will accelerate vaccine development.

## Supporting Information

S1 Fig Amino acid alignment of AM202 for all *A*. *marginale* strains and isolates and *A*. *marginale* ss. *centrale*. AMF_149 is the Florida strain homolog of AM202. ACIS_01081 is the *A*. *marginale* ss. *centrale* ortholog of AM202.
(DOCX)

S2 Fig Amino acid alignment of AM368 for all *A*. *marginale* strains and isolates and *A*. *marginale* ss. *centrale*. AMF_269 is the Florida strain homolog of AM368. ACIS_00938 is the *A*. *marginale* ss. *centrale* ortholog of AM368.
(DOCX)

S3 Fig Amino acid alignment of AM854 for all *A*. *marginale* strains and isolates and *A*. *marginale* ss. *centrale*. AMF_640 is the Florida strain homolog of AM854. ACIS_00486 is the *A*. *marginale* ss. *centrale* ortholog of AM854.
(DOCX)

S4 Fig Amino acid alignment of AM936 for all *A*. *marginale* strains and isolates. **AMF_717 is the Florida strain homolog of AM936.** ACIS_00403 is the *A*. *marginale* ss. *centrale* ortholog of AM936.
(DOCX)

S5 Fig Amino acid alignment of AM1041 for all *A*. *marginale* strains and isolates and *A*. *marginale* ss. *centrale*. N3518.1 and N3518.2 are multiple variants that were obtained from the same isolate. VA.1 and VA.2 are multiple variants that were obtained from the Virginia strain. AMF_790 is the Florida strain homolog of AM1041. ACIS_00314 is the *A*. *marginale* ss. *centrale* ortholog of AM1041.
(DOCX)

S6 Fig Amino acid alignment of AM1096 for all *A*. *marginale* strains and isolates. AMF_828 is the Florida strain homolog of AM1096. ACIS_00268 is the *A*. *marginale* ss. *centrale* ortholog of AM1096.
(DOCX)

S7 Fig A global pairwise alignment of AM854 and APH_0338 (OmpA) using lalign. The line above the alignment (AM854 aa19-68) indicates the binding domain of APH_0338 and denotes the peptide used in the ELISAs. Amino acids that share identity are in white text with a black background. Conservative amino acid replacements are in white text with a gray background.
(PDF)

S8 Fig A global pairwise alignment of AM936 and APH_0248 (Asp14) using lalign. The line above the alignment (AM936 aa84-121) indicates the binding domain of APH_0248 and denotes the peptide used in the ELISAs. Amino acids that share identity are in white text with a black background. Conservative amino acid replacements are in white text with a gray background.
(PDF)

S9 Fig Percent infected erythrocytes over time during challenge. Each bar represents the mean of the percent infected erythrocytes for each group of animals for days 30 to 43 of challenge. The black bars represent the outer membrane (OM) vaccinates, the gray bars represent AM854/AM936 vaccinates (REC), and the white bars represent the adjuvant only vaccinates (ADJ). The error bars are the standard error of the mean. Brackets over the bars indicate groups that are statistically significantly different. The asterisk indicates a p value of <0.05.
(TIF)

S10 Fig Packed cell volume over time during challenge. Each bar represents the mean of the PCV for each group of animals for days 30 to 42 of challenge. The black bars represent the outer membrane (OM) vaccinates, the gray bars represent AM854/AM936 vaccinates (REC), and the white bars represent the adjuvant only vaccinates (ADJ). The error bars are the standard error of the mean. Brackets over the bars indicate groups that are statistically significantly different. The asterisk indicates a p value of <0.05.
(TIF)

S1 Table Oligonucleotides used for sequencing candidate genes. (DOCX)

S2 Table Oligonucleotides used for PCR amplification for cloning and protein expression. (DOCX)

S3 Table Pairwise amino acid identity among all isolates and strains for AM202. (DOCX)

S4 Table Pairwise amino acid identity among all isolates and strains for AM368. (DOCX)

S5 Table Pairwise amino acid identity among all isolates and strains for AM845. (DOCX)

S6 Table Pairwise amino acid identity among all isolates and strains for AM936. (DOCX)

S7 Table Pairwise amino acid identity among all isolates and strains for AM1041. (DOCX)

S8 Table Pairwise amino acid identity among all isolates and strains for AM1096. (DOCX)
