# Supplementary material for: Subdominant Outer Membrane Antigens in Anaplasma marginale: Conservation, Antigenicity, and Protective Capacity Using Recombinant Protein
Source: PLoS One. 2015 Jun 16;10(6):e0129309. doi: 10.1371/journal.pone.0129309 (PMC4469585; doi:10.1371/journal.pone.0129309)
Supplement: S1 Fig — AMF_149 is the Florida strain homolog of AM202. ACIS_01081 is the A. marginale ss. centrale ortholog of AM202. (DOCX) [file pone.0129309.s001.docx]

AM202_6DE 1 MRCRALLLAVLLISTQTGCSLMVAGLAVVTGAVVALQERSVGDVIDDAAILIKINKELFQ
AM202_Dawn 1 MRCRALLLAVLLISTQTGCSLMVAGLAVVTGAVVALQERSVGDVIDDAAILIKINKELFQ
AM202_C51 1 MRCRALLLAVLLISTQTGCSLMVAGLAVVTGAVVALQERSVGDVIDDAAILIKINKELFQ
AM202_C52 1 MRCRALLLAVLLISTQTGCSLMVAGLAVVTGAVVALQERSVGDVIDDAAILIKINKELFQ
AM202_EMΦ 1 MRCRALLLAVLLISTQTGCSLMVAGLAVVTGAVVALQERSVGDVIDDAAILIKINKELFQ
AM202_M18 1 MRCRALLLAVLLISTQTGCSLMVAGLAVVTGAVVALQERSVGDVIDDAAILIKINKELFQ
AM202_M71 1 MRCRALLLAVLLISTQTGCSLMVAGLAVVTGAVVALQERSVGDVIDDAAILIKINKELFQ
AM202_PR 1 MRCRALLLAVLLISTQTGCSLMVAGLAVVTGAVVALQERSVGDVIDDAAILIKINKELFQ
AM202_VA 1 MRCRALLLAVLLISTQTGCSLMVAGLAVVTGAVVALQERSVGDVIDDAAILIKINKELFQ
AM202_StM 1 MRCRALLLAVLLISTQTGCSLMVAGLAVVTGAVVALQERSVGDVIDDAAILIKINKELFQ
AMF_149 1 MRCRALLLAVLLISTQTGCSLMVAGLAVVTGAVVALQERSVGDVIDDAAILIKINKELFQ
ACIS_01081 1 MRCRALLLAVLLISTQTGCSLMVAGLAVVTGAVVALQERSVGDVIDDAAILIKINKELFQ


AM202_6DE 61 QGMFSSITVRVSEGRVLLTGTVDSPDKRLKAERVAWQQSEVKEVVNEIAVDKDEVTLKEV
AM202_Dawn 61 QGMFSSITVRVSEGRVLLTGTVDSPDKRLKAERVAWQQSEVKEVVNEIAVDKDEVTLKEV
AM202_C51 61 QGMFSSITVRVSEGRVLLTGTVDSPDKRLKAERVAWQQSEVKEVVNEIAVDKDEVTLKEV
AM202_C52 61 QGMFSSITVRVSEGRVLLTGTVDSPDKRLKAERVAWQQSEVKEVVNEIAVDKDEVTLKEV
AM202_EMΦ 61 QGMFSSITVRVSEGRVLLTGTVDSPDKRLKAERVAWQQSEVKEVVNEIAVDKDEVTLKEV
AM202_M18 61 QGMFSSITVRVSEGRVLLTGTVDSPDKRLKAERVAWQQSEVKEVVNEIAVDKDEVTLKEV
AM202_M71 61 QGMFSSITVRVSEGRVLLTGTVDSPDKRLKAERVAWQQSEVKEVVNEIAVDKDEVTLKEV
AM202_PR 61 QGMFSSITVRVSEGRVLLTGTVDSPDKRLKAERVAWQQSEVKEVVNEIAVDKDEVTLKEV
AM202_VA 61 QGMFSSITVRVSEGRVLLTGTVDSPDKRLKAERVAWQQSEVKEVVNEIAVDKDEVTLKEV
AM202_StM 61 QGMFSSITVRVSEGRVLLTGTVDSPDKRLKAERVAWQQSEVKEVVNEIAVDKDEVTLKEV
AMF_149 61 QGMFSSITVRVSEGRVLLTGTVDSPDKRLKAERVAWQQSEVKEVVNEIAVDKDEVTLKEV
ACIS_01081 61 HGIFSSITVRVSEGRVLLTGTVDSPDKRLKAERVAWQQSEVKEVVNEIAVDKDEVTLKEV


AM202_6DE 121 AIDSAISAQIKARMVAHAGIKSVNYSINTVGGVVYLMGIAQSQKELNSVIGISKRVKGVK
AM202_Dawn 121 AIDSAISAQIKARMVAHAGIKSVNYSINTVGGVVYLMGIAQSQKELNSVIGISKRVKGVK
AM202_C51 121 AIDSAISAQIKARMVAHAGIKSVNYSINTVGGVVYLMGIAQSQKELNSVIGISKRVKGVK
AM202_C52 121 AIDSAISAQIKARMVAHAGIKSVNYSINTVGGVVYLMGIAQSQKELNSVIGISKRVKGVK
AM202_EMΦ 121 AIDSAISAQIKARMVAHAGIKSVNYSINTVGGVVYLMGIAQSQKELNSVIGISKRVKGVK
AM202_M18 121 AIDSAISAQIKARMVAHAGIKSVNYSINTVGGVVYLMGIAQSQKELNSVIGISKRVKGVK
AM202_M71 121 AIDSAISAQIKARMVAHAGIKSVNYSINTVGGVVYLMGIAQSQKELNSVIGISKRVKGVK
AM202_PR 121 AIDSAISAQIKARMVAHAGIKSVNYSINTVGGVVYLMGIAQSQKELNSVIGISKRVKGVK
AM202_VA 121 AIDSAISAQIKARMVAHAGIKSVNYSINTVGGVVYLMGIAQSQKELNSVIGISKRVKGVK
AM202_StM 121 AIDSAISAQIKARMVAHAGIKSVNYSINTVGGVVYLMGIAQSQKELNSVIGISKRVKGVK
AMF_149 121 AIDSAISAQIKARMVAHAGIKSVNYSINTVGGVVYLMGIAQSQKELNSVIGISKRVKGVK
ACIS_01081 121 AIDSAISAQIKARMVAHAGIKSVNYSINTVGGVVYLMGIAQSQKELNSVIGISKRVKGVK


AM202_6DE 181 QVISYVRLKHSKLRR
AM202_Dawn 181 QVISYVRLKHSKLRR
AM202_C51 181 QVISYVRLKHSKLRR
AM202_C52 181 QVISYVRLKHSKLRR
AM202_EMΦ 181 QVISYVRLKHSKLRR
AM202_M18 181 QVISYVRLKHSKLRR
AM202_M71 181 QVISYVRLKHSKLRR
AM202_PR 181 QVISYVRLKHSKLRR
AM202_VA 181 QVISYVRLKHSKLRR
AM202_StM 181 QVISYVRLKHSKLRR
AMF_149 181 QVISYVRLKHSKLRR
ACIS_01081 181 QVISYVRLKHSKLRR

Fig. S1. Amino acid alignment of AM202 for all *A. marginale* strains and isolates and *A. marginale* ss. *centrale*. AMF_149 is the Florida strain homolog of AM202. ACIS_01081 is the *A. marginale* ss. *centrale* ortholog of AM202.
