# Supplementary material for: Subdominant Outer Membrane Antigens in Anaplasma marginale: Conservation, Antigenicity, and Protective Capacity Using Recombinant Protein
Source: PLoS One. 2015 Jun 16;10(6):e0129309. doi: 10.1371/journal.pone.0129309 (PMC4469585; doi:10.1371/journal.pone.0129309)
Supplement: S2 Fig — AMF_269 is the Florida strain homolog of AM368. ACIS_00938 is the A. marginale ss. centrale ortholog of AM368. (DOCX) [file pone.0129309.s002.docx]

AM368_6DE 1 MLSRNVRNGHQFASVNCTHTWYGYNGTTPVYRWLLRGHVVSSAWTTSRSYGIAHISAYGA
AM368_Dawn 1 MLSRNVRNGHQFASVNCTHTWYGYNGTTPVYRRLLRGHVVSSAWTTSRSYGIAHISAYGA
AM368_C51 1 MLSRNVRNGHQFASVNCTHTWYGYNGTTPVYRWLLRGHVVSSAWTTSRSYGIAHISAYGA
AM368_C52 1 MLSRNVRNGHQFASVNCTHTWYGYNGTTPVYRWLLQGHVVSSAWTTSRSYGIAHISAYGA
AM368_EMΦ 1 MLSRNVRNGHQFASVNCTHTWYGYNGTTPVYRWLLQGHVVSSAWTTSRSYGIAHISAYGA
AM368_N3518 1 MLSRNVRNGHQFASVNCTHTWYGYNGTTPVYRWLLRGHVVSSAWTTSRSYGIAHISAYGA
AM368_N3571 1 MLSRNVRNGHQFASVNCTHTWYGYNGTTPVYRWLLRGHVVSSAWTTSRSYGIAHISAYGA
AM368_PR 1 MLSRNVRNGHQFASVNCTHTWYGYNGTTPVYRWLLRGHVVSSAWTTSRSYGIAHISAYGA
AM368_VA 1 MLSRNVRNGHQFASVNCTHTWYGYNGTTPVYRWLLQGHVVSSAWTTSRSYGIAHISAYGA
AM368_StM 1 MLSRNVRNGHQFASVNCTHTWYGYNGTTPVYRWLLRGHVVSSAWTTSRSYGIAHISAYGA
AMF_269 1 MLSRNVRNGHQFASVNCTHTWYGYNGTTPVYRWLLQGHVVSSAWTTSRSYGIAHISAYGA
ACIS_00938 1 LLSV-----------------VGGRGSAVRLLALLPGREKKSAAQPRQ----------GA


AM368_6DE 61 AHLPLGIGRSPVHNNNEDTTQT-DGSGEPSAEEGRDGCASSPE-----IGTSEGSSEAPE
AM368_Dawn 61 AHLPLGIGRSPVHNNNEDTTQT-DGSGEPSAEEGRDGCASSPE-----IGTSEGSSEAPE
AM368_C51 61 AHLPLGIGRSPVHNNNEDTTQT-DGSGEPSAEEGRDGCASSPE-----IGTSEGSSEAPE
AM368_C52 61 AHLPLGIGRSPVHNNNEDTTQT-DGSGEPSAEEGRDGCASSPE-----IGTSEGSSEAPE
AM368_EMΦ 61 AHLPLGIGRSPVHNNNEDTTQT-DGSGEPSAEEGRDGCASSPE-----IGTSEGSSEAPE
AM368_N3518 61 AHLPLGIGRSPVHNNNEDTTQT-DGSGEPSAEEGRDGCASSQE-----IGTSEGSSEAPE
AM368_N3571 61 AHLPLGIGRSPVHNNNEDTTQT-DGSGEPSAEEGRDGCASSPE-----IGTSEGSSEAPE
AM368_PR 61 AHLPLGIGRSPVHNNNEDTTQT-DGSGEPSAEEGRDGCASSQE-----IGTSEGSSEAPE
AM368_VA 61 AHLPLGIGRSPVHNNNEDTTQT-DGSGEPSAEEGRDGCASSPE-----IGTSEGSSEAPE
AM368_StM 61 AHLPLGIGRSPVHNNNEDTTQT-DGSGEPSAEEGRDGCASSQE-----IGTSEGSSEAPE
AMF_269 61 AHLPLGIGRSPVHNNNEDTTQT-DGSGEPSAEEGRDGCASSPE-----IGTSEGSSEAPE
ACIS_00938 34 AGS--GSNCSTTSSNLPDNEQAQNGSASPS--EQNEGCTHATHGAVGSVGSFFSDSVANS


AM368_6DE 115 VEGTTHSAAPATPALEL-------KSAPDQEMCLAAPTPWSLIYSAKHHDDN-NAAQYTH
AM368_Dawn 115 VEGTTHSAAPATPALEL-------KSAPDQEMCLAAPTPWSLIYSAKHHDDN-KAAQYEH
AM368_C51 115 VEGTTHSAAPATPALEL-------KSAPDQEMCLAAPTPWSLIYSAKHHDDN-KAAQYEH
AM368_C52 115 VEGTTHSAAPATPALEL-------KSAPDQEMCLAAPTPWSLIYSAKHHDDN-KAAQYEH
AM368_EMΦ 115 VEGTTHSAAPATPALEL-------KSAPDQEMCLAAPTPWSLIYSAKHHDDN-KAAQYKR
AM368_N3518 115 VEGTTHSAAPATPALEL-------KSAPDQEMCLAAPTPWSLIYSAKHHDDN-KAAQYEH
AM368_N3571 115 VEGTTHSAAPATPALEL-------KSAPDQEMCLAAPTPWSLIYSAKHHDDN-KAAQYEH
AM368_PR 115 VEGTTHSAAPATPALEL-------KSAPDQEMCLAAPTPWSLIYSAKHHDDN-KAAQYEH
AM368_VA 115 VEGTTHSAAPATPALEL-------KSAPDQEMCLAAPTPWSLIYSAKHHDDN-KAAQYEH
AM368_StM 115 VEGTTHSAAPATPALEL-------KSAPDQEMCLAAPTPWSLIYSAKHHDDN-KAAQYEH
AMF_269 115 VEGTTHSAAPATPALEL-------KSAPDQEMCLAAPTPWSLIYSAKHHDDN-KAAQYEH
ACIS_00938 90 REDVRDDTLPSSGSAELAATAPTLKTRVDQKMRDAAPEPWSLICASTHDSCGISSIPQND


AM368_6DE 167 VQDVCSQYLCKHAKDRHGLLKFIAASIDKGVSASIDVAANTTRASMRAIRNYKTE-IVAA
AM368_Dawn 167 VQDVCSQYLCKHAKDRHGLLKFIAASIDKGVSASIDVAANTTRASMRAIRNYKTE-IVAA
AM368_C51 167 VQDVCSQYLCKHAKDRHGLLKFIAASIDKGVSASIDVAANTTRASMRAIRNYKTE-IVAA
AM368_C52 167 VQDVCSQYLCKHAKDRHGLLKFIAASIDKGVSASIDVAANTTRASMRAIRNYNTE-IVAA
AM368_EMΦ 167 VQDVCSQYLCKHAKDRHGLLKFIAASIDKGVSASIDVAANTTRASMRAIRNYKTE-IVAA
AM368_N3518 167 VQDVCSQYLCKHAKDRHGLLKFIAASIDKGVSASIDVAANTTRASMRAIRNYKTE-IVAA
AM368_N3571 167 VQDVCSQYLCKHAKDRHGLLKFIAASIDKGVSASIDVAANTTRASMRAIRNYKTE-IVAA
AM368_PR 167 VQDVCRQYLCKHAKDRHGLLKFIAASIDKGVSASIDVAANTTRASMRAIRNYETEEIVAA
AM368_VA 167 VQDVCSQYLCKHAKDRHGLLKFIAASIDKGVSASIDVAANTTRASMRAIRNYKTE-IVAA
AM368_StM 167 VQDVCSQYLCKHAKDRHGLLKFIAASIDKGVSASIDVAANTTRASMRAIRNCKTE-IVAA
AMF_269 167 VQDVCSQYLCKHAKDRHGLLKFIAASIDKGVSASIDVAANTTRASMRAIRNDKTE-IVAA
ACIS_00938 150 IREFCRKYLYQKPKDRQGLINFIRASITNISTTSIISVASTEKASMRTIHSSSDDSIAGM

AM368_6DE 226 KITEKHTFTVKS-AGSDIGALAADMKYIVRPTPQPCKYKICDLEVTIRHSDGAE--LTYT
AM368_Dawn 226 KITEKHTFTVKS-AGSDICALAADMKYIVRPTPQPCKYKICDLEVTIRHSDGAE--LTYT
AM368_C51 226 KITEKHTFTVKS-AGSDIGALAADMKYIVRPTPQPCKYKICDLEVTIRHSDGAE--LTYT
AM368_C52 226 KITEKHTFTVKS-AGSDIGALAADMKYIVRPTPQPCKYKICDLEVTIRHSDGAE--LTYT
AM368_EMΦ 226 KITEKHTFTVKS-AGSDIGALAADMKYIVRPTPQPCKYKICDLEVTIRHSDGAE--LTYT
AM368_N3518 226 KITEKHTFTVKS-AGSDIGALAADMKYIVRPTPQPCKYKICDLEVTIRHSDGAE--LTYT
AM368_N3571 226 KITEKHTFTVKS-AGSDIGALAADMKYIVRPTPQPCKYKICDLEVTIRHSDGAE--LTYT
AM368_PR 227 KITEKHTFTVKS-AGSDIGALAADMKYIVRPTPQPCKYKICDLEVTIRHSDGAE--LTYT
AM368_VA 226 KITEKHTFTVKS-AGSDIGALAADMKYIVRPTPQPCKYKICDLEVTIRHSDGAE--LTYT
AM368_StM 226 KITEKHTFTVKS-AGSDIGALAADMKYIVRPTPQPCKYKICDLEVTIRHSDGAE--LTYT
AMF_269 226 KITEKHTFTVKS-AGSDIGALAADMKYIVRPTPQPCKYKICDLEVTIRHSDGAE--LTYT
ACIS_00938 210 KVTEQHTFYVQSSANGHIHVLVADIEYKVRPTSTQNRYKICDLSIEIRR-DGVNTRLLYS

AM368_6DE 283 STNISEVPSSGAQNTVPAQSVDDVDDNAQSVTPDADLATPSDDTYSQSAELTQSAEFTTT
AM368_Dawn 283 STNISEVPSSGAQNTVPAQSVDDVDDNAQSVTPDADLATPSDDTYSQSAELTQSAEFTTT
AM368_C51 283 STNISEVPSSGAQNTVPAQSVDDVDDNAQSVTPDADLATPSDDTYSQSAELTQSAEFTTT
AM368_C52 283 STNISEVPSSGAQNTVPAQSVDDVDDNAQSVTPDADLATPSDDTYSQSAELTQSAEFTTT
AM368_EMΦ 283 STNISEVPSSGAQNTVPAQSVDDVDDNAQSVTPDADLATPSDDTYSQSAELTQSAEFTTT
AM368_N3518 283 STNISEVPSSGAQNTVPAQSVDDVDDNAQSVTPDADLATPSDDTYSQSAE------FTTT
AM368_N3571 283 STNISEVPSSGAQNTVPAQSVDDVDDNAQSVTPDADLATPSDDTYSQSAELTQSAEFTTT
AM368_PR 284 STNISEVPSSGAQNTVPAQSVDDVDDNAQSVTPDADLATPSDDTYSQSAELTQSAEFTTT
AM368_VA 283 STNISEVPSSGAQNTVPAQSVDDVDDNAQSVTPDADLATPSDDTYSQSAELTQSAEFTTT
AM368_StM 283 STNISEVPSSGAQNTVPAQSVDDVDDNAQSVTPDADLATPSDDTYSQSAE------FTTT
AMF_269 283 STNISEVPSSGAQNTVPAQSVDDVDDNAQSVTPDADLATPSDDTYSQSAELTQSAEFTTT
ACIS_00938 269 CNGTSEV-------SLPITRA--AHRGAQNATSDTH--SDASDSCGAGGEL---------


AM368_6DE 343 QDVDVAPDAENADTQQTRNSFISPPEAAPSDEIDVPKDRYVTALAASMRTVREVIISLCN
AM368_DAWN 343 QDVDVAPDAENADTQQTRNSFISPPEAAPSDEIDVPKDRYVTALAASMRTVREVIISLCN
AM368_C51 343 QDVDVAPDVENADTQQTRNSFISPPEAAPSDEIDVPKDRYVTALAASMRTVREVIISLCN
AM368_C52 343 QDVDVAPDAENADTQQTRNSFISPPEAAPSDEIDVPKDRYVTALAASMRTVREVIISLCN
AM368_EMΦ 343 QDVDVAPDAENADTQQTRNSFISPPEAAPSDEIDVPKDRYVTALAASMRTVREVIISLCN
AM368_N3518 337 QDVDVAPDAENADTQQTRNSFISPPEAAPSDEIDVPKDRYVTALAASMRTVREVIISLCN
AM368_N3571 343 QDVDVAPDVENADTQQTRNSFISPPEAAPSDEIDVPKDRYVTALAASMRAVRDVIVSLHN
AM368_PR 344 QDVDVAPDVENADAQQTRNSFISPPEAAPSDEIDVPKDRYVTALAASMRTVREVIISLCN
AM368_VA 343 QDVDVAPDVENADTQQTRNSFISPPEAAPSDEIDVPKDRYVTALAASMRTVREVIISLCN
AM368_StM 337 QDVDVAPDAENADTQQTRNSFISPPEAAPSDEIDVPKDRYVTALAASMRTVREVIISLCN
AMF_269 343 QDVDVAPDVENADTQQTRNSFISPPEAAPSDEIDVPKDRYVTALAASMRTVREVIISLCN
ACIS_00938 309 ----ITPDVV-AETQK--------PSSA--------------------------------


AM368_6DE 403 NTQHLADPSEDVLGSDAIPNCTTVRISPKLVVKAYGEFIREMLNDPATVGDNIPNAAEQE
AM368_Dawn 403 NTQHLADPSEDVLGSDAIPNCTTVRISPKLVAKAYGEFIREMLNDPAIVGDNTPNAAEQE
AM368_C51 403 NTQHLADPSEDVLGSDAIPNCTTVRISPKLVVKAYGEFIREMLNDPATVGDNIPNAAEQE
AM368_C52 403 NTQHLADPSEDVLGSDAIPNCTTVRISPKLVVKAYGEFIRKMLNDPATVGDNIPNAAEQE
AM368_EMΦ 403 NTQHFADPSEDVLGSDAIPNCTTVRISPKLVVKAYGEFIREMLNDPATVGDNIPNAAEQE
AM368_N3518 397 NTQHLADPSEDVLGSDAIPNCTTVRISPKLVVKAYGEFIREMLNDPATVGDNIPNAAEQE
AM368_N3571 403 NTQHLADPSEDVLVSDAIPNCTTVRISPKLVVKAYGEFIREMLNDPAIVGDNTPNAAEQE
AM368_PR 404 NTQHLADPSEDVLGSDAIPNCTTVRISPKLVVKAYGEFIREMLNDPAIVGDNTPNAAEQE
AM368_VA 403 NTQHLADPSEDVLGSDAIPNCTTVRISPKLVAKAYGEFIREMLNDPAIVGDNIPNAAEQE
AM368_StM 397 NTQHSADPSEDVLGSDAIPNCTTVRISPKLVAKAYGEFIREMLNDPAIVGDNTPNAAEQE
AMF_269 403 NTQHLADPSEDVLGSDAIPNCTTVRISPKLVAKAYGEFIREMLNDPAIVGDNIPNAAEQE
ACIS_00938 324 ---HAAEAGQE-------------------------------------------------

AM368_6DE 463 KIDCIHNHAQYIVTTVIEAAQGATGVHE---NDIAQTVRQKILAELAPDIPTTDAMIEPV
AM368_Dawn 463 KIDDIHNHAQYIVTTVIEAAQGKTGVHE---NDIAQTVRQKILAELAPDIPTTDAMIEPV
AM368_C51 463 KIDDIHNHAQYIVQTVIEAAQGETGVHE---NDIAQTVRQKILAELAPDIPTTDAMIEPV
AM368_C52 463 KIDCIHNHAQYIVTTVIEAAQGATGVHE---NDIAQTVRQKILAELAPDIPTTDAMIEPV
AM368_EMΦ 463 KIDCIHNHAQYIVTTVIEAAQGATGVHE---NDIAQTVRQKILAELAPDIPTTDAMIEPV
AM368_N3518 457 KIDDIHNHAQYIVQTVIEAAQGETGVHE---NDIAQTVRQKILAELAPDIPTTDAMIEPV
AM368_N3571 463 KIDDIHNHAQYIVQTVIEAAQGETGVHE---NDIAQTVRQKILAELAPDIPTTDAMIEPV
AM368_PR 464 KIDDIHNHAQYIVTTVIEAAQGKTGVHE---NDIAQTVRQKILAELAPDIPTTDAMTEPV
AM368_VA 463 KIDCIHNHAQYIVTTVIEAAQGATGVHE---NDIAQTVRQKILAELAPDIPTTDAMIEPV
AM368_StM 457 KIDDIHNHAQYIVTTVIEAAQGATGVHE---NDIAQTVRQKILAELAPDIPTTDAMIEPV
AMF_269 463 KIDCIHNHAQYIVTTVIEAAQGATGVHE---NDIAQTVRQKILAELAPDIPTTDAMIEPV
ACIS_00938 332 --------------------QGESGVSEASGNAHAETPRAR-------------------


AM368_6DE 520 QDEHVDITPDSEEQETMYENGK-THPDRSLPTE-PVFTMPFWSAAMFFASAMMGICDLLQ
AM368_Dawn 520 QDEHVDITPDSEEQETMYENDKKTHPDRSLPTE-PVFTMPFWSAAMFFASAMMGICDLLQ
AM368_C51 520 QDEHVDITPDSEEQETMYENDKKTHPDRSLPTEEPVFTMPFWSAAMFFASAMMGICDLLQ
AM368_C52 520 QDEHVDITPDSEEQETMYENGK-THPDRSLPTE-PVFTMPFWSAAMFFASAMMGICDLLQ
AM368_EMΦ 520 QDEHVDITPDSEEQETMYENGK-THPDRSLPTE-PVFTMPFWSAAMFFASAMMGICDLLQ
AM368_N3518 514 QDEHVDITPDSEEQETMYENDKKTHPDRSLPTEEPVFTMPFWSAAMFFASAMMGICDLLQ
AM368_N3571 520 QDEHVDITPDSEEQETMYENDKKTHPDRSLPTEEPVFTMPFWSAAMFFASAMMGICDLLQ
AM368_PR 521 QDEHVDITPDSEEQETMYENDKKTHPDRSLPTE-PVFTMPFWSAAMFFASAMMGICDLLQ
AM368_VA 520 QDEHVDITPDSEEQETMYENGK-THPDRSLPTE-PVFTMPFWSAAMFFASAMMGICDLLQ
AM368_StM 514 QDEHVDITPDSEEQETMYENGK-THPDRSLPTE-PVFTMPFWSAAMFFASAMMGICDLLQ
AMF_269 520 QDEHVDITPDSEEQETMYENGK-THPDRSLPTE-PVFTMPFWSAAMFFASAMMGICDLLQ
ACIS_00938 353 ------------------------------------------------------------

AM368_6DE 578 QEYEIELITNVEISRLVANLLASSRKDNNVAISEARIREVFTACQQEISEKFLSCTGQEF
AM368_Dawn 579 QEYEIELITNVEISRLVANLLASSRKDNNVAISEARIRDVFTACQQEISEKFLSCTGQEF
AM368_C51 580 QEYEIELITNVEISRLVANLLASSRKDNNVAISEARIRDVFTACQQEISEKFLSCTGQEF
AM368_C52 578 QEYEIELITNVEISRLVANLLASSRKDNNVAISEARIREVFTACQQEISEKFLSCTGQEF
AM368_EMΦ 578 QEYEIELITNVEISRLVANLLASSRKDNNVAISEARIREVFTACQQEISEKFLSCTGQEF
AM368_N3518 574 QEYEIELITNVEISRLVANLLASSRKDNNVAISEARIRDVFTACQQEISEKFLSCTGQEF
AM368_N3571 580 QEYEIELITNVEISRLVANLLASSRKDNNVAISEARIRDVFTACQQEISEKFLSCTGQEF
AM368_PR 580 QEYEIELITNVEISRLVANLLASSRKDNNVAISEARIREVFTACQQEISEKFLSCTGQEF
AM368_VA 578 QEYEIELITNVEISRLVANLLASSRKDNNVAISEARIREVFTACQQEISEKFLSCTGQEF
AM368_StM 572 QEYEIELITNVEISRLVANLLASSRKDNNVAISEARIREVFTACQQEISEKFLSCTGQEF
AMF_269 578 QEYEIELITNVEISRLVANLLASSRKDNNVAISEARIREVFTACQQEISEKFLSCTGQEF
ACIS_00938 353 --------------------------------------------GSEIRRESLTSTDEEH


AM368_6DE 638 ECDSR-FIQQMTSIVLKVVESYNGVSLTDWSVTDKLKQKAAKLFVRYHADLFEGIQPNEG
AM368_Dawn 639 ECDSR-FIQQMTSIVLKVVESYDGVSLTDWSVTDKLKQKAAKLFVRYHADLFEGIQPNEG
AM368_C51 640 ECDSR-FIQQMTSIVLKVVESYDGVSLTDWSVTDKLKQKAAKLFVRYHADLFEGIQPNEG
AM368_C52 638 ECDSR-FIQQMTSIVLKVVESYDGVSLTDWSVTDKLKQKAAKLFVRYHADLFEGIQPNEG
AM368_EMΦ 638 ECDSR-FIQQMTSIVLKVVESYDGVSLTDWSVTDKLKQKAAKLFVRYHADLFEGIQPNEG
AM368_N3518 634 ECDSR-FIQQMTSIVLKVVESYDGVSLTDWSVTDKLKQKAAKLFVRYHADLFEGIQPNEG
AM368_N3571 640 ECDSR-FIQQMTSIVLKVVESYDGVSLTDWSVTDKLKQKAAKLFVRYHADLFEGIQPNEG
AM368_PR 640 ECDSR-FIQQMTSIVLKVVESYNGVSLTDWSVTDKLKQKAAKLFVRYHADLFEGIQPNEG
AM368_VA 638 ECDSR-FIQQMTSIVLKVVESYDGVSLTDWSVTDKLKQKAAKLFVRYHADLFEGIQPNEG
AM368_StM 632 ECDSR-FIQQMTSIVLKVVESYNGVSLTDWSVTDKLKQKAAKLFVRYHADLFEGIQPNEG
AMF_269 638 ECDSR-FIQQMTSIVLKVVESYDGVSLTDWSVTDKLKQKAAKLFVRYHADLFEGIQPNEG
ACIS_00938 369 QHDQPLFADTIAQAVLQTAGRRGSASSTDGSVTETFRQRAYESFPDY------GVQHTLA

AM368_6DE 697 PEPLQLGQQLGNCNAAEAALLWSGTEFKLANDVTAILGDTPWVKHLFFAQPKSNCGLAEH
AM368_Dawn 698 PEPLQLGKQLGNCNAAEAAFLWSGTEFKLANDVTAILGDTPWVKHLFFAQPKSNCGLAEH
AM368_C51 699 PEPLQLGKQLGNCNAAEAAFLWSGTEFKLANDVTAILGDTPWVKHLFFAQPKSNCGLAEH
AM368_C52 697 PEPLQLGQQLGNCNAAEAALLWSGTEFKLANDVTAILGDTPWVKHLFFAQPKSNCGLAEH
AM368_EMΦ 697 PEPLQLGQQLGNCNAAEAALLWSGTEFKLANDVTAILGDTPWVKHLFFAQPKSNCGLAEH
AM368_N3518 693 PEPLQLGKQLGNCNAAEAAFLWSGTEFKLANDVTAILGDTPWVKHLFFAQPKSNCGLAEH
AM368_N3571 699 PEPLQLGKQLGNCNAAEAAFLWSGTEFKLANDVTAILGDTPWVKHLFFAQPKSNCGLAEH
AM368_PR 699 PEPLQLGQQLGNCNAAEAALLWSGTEFKLANDVTAILGDTPWVKHLFFAQPKSNCGLAEH
AM368_VA 697 PEPLQLGKQLGNCNAAEAAFLWSGTEFKLANDVTAILGDTPWVKHLFFAQPKSNCGLAEH
AM368_StM 691 PEPLQLGQQLGNCNAAEAALLWSGTEFKLANDVTAILGDTPWVKHLFFAQPKSNCGLAEH
AMF_269 697 PEPLQLGKQLGNCNAAEAAFLWSGTEFKLANDVTAILGDTPWVKHLFFAQPKSNCGLAEH
ACIS_00938 423 PETLQSSDQSGTANATVAASLWSGTEFTLAKDVEEILCDEHFVQNLFFAQQGEASSFAKK


AM368_6DE 757 LILGIIKRIAEQSSLPGSDFKIEGKISSRSRLRKGGSYHVTHKATVAHIHSHEE---IGV
AM368_Dawn 758 LILGIIKRITERSSLAGSDFKIEGKISSRSRLRKGGSYHVTHKATVAHIHSHEE---IGV
AM368_C51 759 LILGIIKRITERSSLAGSDFKIEGKISSRSRLRKGGSYHVTHKATVAHIHSHEE---IGV
AM368_C52 757 LILGIIKRIAEQSSLPGSDFKIEGKISSRSRLRKGGSYHVTHKATVAHIHSHEE---IGV
AM368_EMΦ 757 LILGIIKRITERSSLAGSDFKIEGKISSRSRLRKGGSYHVTHKATVTHIQSHEE---IGV
AM368_N3518 753 LILGIIKRIAEQSSLPGSDFKIEGKISSRSRLRKGGSYHVTHKATVAHIHSHEE---IGV
AM368_N3571 759 LILGIIKRITERSSLAGSDFKIEGKISSRSRLRKGGSYHVTHKATVAHIHSHEE---IGV
AM368_PR 759 LILGIIKRIAEQSSLPGSDFKIEGKISSRSRLRKGGSYHVTHKATVAHIHSHEE---IGV
AM368_VA 757 LILGIIKRITERSSLAGSDFKIEGKISSRSRLRKGGSYHVTHKATVAHIHSHEE---IGV
AM368_StM 751 LILGIIKRITERSSLAGSDFKIEGKISSRSRLRKGGSYHVTHKATVAHIHSHEE---IGV
AMF_269 757 LILGIIKRITERSSLAGSDFKIEGKISSRSRLRKGGSYHVTHKATVAHIHSHEE---IGV
ACIS_00938 483 LILGIFEKLAKRS-LPDSDFKIEDEVLSRSRLKKEGSYRVTHKATMVRTRDREEAEKMRV


AM368_6DE 814 YLSYTVHPSKSANRPKYRITNPKLSVLGTDPEQVACFKIENMASKDSLPFTDAEAMY-NK
AM368_Dawn 815 YLSYTVHPSKSANRPKYRITNPKLSVLGTDPEQVACFKIENMASKDSLPFTDAEAMY-NK
AM368_C51 816 YLSYTVHPSKSANRPKYRITNPKLSVLGTDPEQVACFKIENMASKDSLPFTDAEAMY-NK
AM368_C52 814 YLSYTVHPSKSANRPKYRITNPKLSVLGTDPEQVACFKIENMASKDSLPFTDAEAMY-NK
AM368_EMΦ 814 YLSYTVHPSKSANRPKYRITNPKLSVLGTDPEQVACFKIENMASKDSLPFTDAEAMY-NK
AM368_N3518 810 YLSYTVHPSKSANRPKYRITNPKLSVLGTDPEQVACFKIENMASKDSLPFTDAEAMY-NK
AM368_N3571 816 YLSYTVHPSKSANRPKYRITNPKLSVLGTDPEQVACFKIENMASKDSLPFTDAEAMY-NK
AM368_PR 816 YLSYTVHPSKSANRPKYRITNPKLSVLGTDPEQVACFKIENMASKDSLPFTDAEAMY-NK
AM368_VA 814 YLSYTVHPSKSANRPKYRITNPKLSVLGTDPEQVACFKIENMASKDSLPFTDAEAMY-NK
AM368_StM 808 YLSYTVHPSKSANRPKYRITNPKLSVLGTDPEQVACFKIENMASKDSLPFTDAEAMY-NK
AMF_269 814 YLSYTVHPSKSANRPKYRITNPKLSVLGTDPEQVACFKIENMASKDSLPFTDAEAMY-NK
ACIS_00938 542 HLRYTVRLNPTTDKP---------SVPGTHREQVQCLNTEDMASNSSIPPADAQNLHDNS

AM368_6DE 873 TREKRSQHKHGDLQTQQAPTKEKHQKILGGICAALKAILCAIWKFITYPFTLLASCLSFK
AM368_Dawn 874 TREKRSQHKHGDLQTQQAPTKEKHQKILGGICAALKAILCAIWKFITYPFTLLASCLSFK
AM368_C51 875 TREKRSQHKHGDLQTQQAPTKEKHQKILVGICAALKAILCAIWKFITYPFTLLASCLSFK
AM368_C52 873 TREKRSQHKHGDLQTQQAPTKEKHQKILGGICAALKAILCAIWKFITYPFTLLASCLSFK
AM368_EMΦ 873 TREKRSQHKHGDLQTQQAPTKEKHQKILGGICAALKAILCAIWKFITYPFTLLASCLSFK
AM368_N3518 869 TREKRSQHKHGDLQTQQAPTKEKHQKILVGICAALKAILCAIWKFITYPFTLLASCLSFK
AM368_N3571 875 TREKRSQHKHGDLQTQQAPTKEKHQKILGGICAALKAILCAIWKFITYPFTLLASCLSFK
AM368_PR 875 TREKRSQHKHGDLQTQQAPTKEKHQKILGGICAALKAILCAIWKFITYPFTLLASCLSFK
AM368_VA 873 TREKRSQHKHGDLQTQQAPTKEKHQKILGGICAALKAILCAIWKFITYPFTLLASCLSFK
AM368_StM 867 TREKRSQHKHGDLQTQQAPTKEKHQKILVGICAALKAILCAIWKFITYPFTLLASCLSFK
AMF_269 873 TREKRSQHKHGDLQTQQAPTKEKHQKILGGICAALKAILCAIWKFITYPFTLLASCLSFK
ACIS_00938 593 MRGGCNKSKPASAQEEEA--RSLLAKIFCRVYAAFRAILRTIWKFIVYPFTLLVSRISPN

AM368_6DE 933 RHGSQTTSDNAAVTTENLAHRHPQAHSSSRGNH-RRARHK-AQSSREEQRIQVRHSRPDR
AM368_Dawn 934 RHGSQTTSDNAAVTTENLAHRHPQAHSSSRGNH-RRARHK-AQSSREEQRIQVRHSRPDR
AM368_C51 935 RHGSQTTSDNVAVTTENLAHRHPQAHSSSRGNH-RRARHK-AQSSREEQRIQVRHSRPDR
AM368_C52 933 RHGSQTTSDNVAVTTENLAHRHPQAHSSSRGNH-RRARHK-AQSSREEQRIQVRHSRPDR
AM368_EMΦ 933 RHGSQTTSDNAAVTTENLAHRHPQAHSSSRGNH-RRARHK-AQSSREEQRIQVRHSRPDR
AM368_N3518 929 RHGSQTTSDNAAVTTENLAHRHPQAHSSSRGNH-RRARHK-AQSSREEQRIQVRHSRPDR
AM368_N3571 935 RHGSQTTSDNAAVTTENLAHRHPQAHSSSRGNH-RRARHK-AQSSREEQRIQVRHSRPDR
AM368_PR 935 RHGSQTTSDNAAVTTENLAHRHPQAHSSSRGNH-RRARHK-AQSSREEQRIQVRHSRPDR
AM368_VA 933 RHGSQTTSDNAAVTTENLAHRHPQAHSSSRGNH-RRARHK-AQSSREEQRIQVRHSRPDR
AM368_StM 927 RHGSQTTSDNVAVTTENLAHRHPQAHSSSRGNH-RRARHK-AQSSREEQRIQVRHSRPDR
AMF_269 933 RHGSQTTSDNAAVTTENLAHRHPQAHSSSRGNH-RRARHK-AQSSREEQRIQVRHSRPDR
ACIS_00938 651 RNSSNITSDDVVVTEKSPGS---QIHSSSRGSHAHRSQHEVAEGMREKRNPATGHSDLER


AM368_6DE 991 KADIDD-APTTVMSNIEVNSLSIPMARGTF
AM368_Dawn 992 KADIDD-APTTVMSNIEVNSLSIPMARGTF
AM368_C51 993 KADIDD-APTTVMSNIEVNSLSIPMARGTF
AM368_C52 991 KADIDD-APTTVMSNIEVNSLSIPMARGTF
AM368_EMΦ 991 KADIDD-APTTVMSNIEVNSLSIPMARGTF
AM368_N3518 987 KADIDD-APTTVMSNIEVNSLSIPMARGTF
AM368_N3571 993 KADIDD-APTTVMSNIEVNSLSIPMARGTF
AM368_PR 993 KADIDD-APTTVMSNIEVNSLSIPMARGTF
AM368_VA 991 KADIDD-APTTVMSNIEVNSLSIPMARGTF
AM368_StM 985 KADIDD-APTTVMSNIEVNSLSIPMARGTF
AMF_269 991 KADIDD-APTTVMSNIEVNSLSIPMARGTF
ACIS_00938 708 KGRTDSGSPSTAMSDVEVNSLRSHANLRSA


Fig. S2. Amino acid alignment of AM368 for all *A. marginale* strains and isolates and *A. marginale* ss. *centrale*. AMF_269 is the Florida strain homolog of AM368. ACIS_00938 is the *A. marginale* ss. *centrale* ortholog of AM368.
