# Supplementary material for: Subdominant Outer Membrane Antigens in Anaplasma marginale: Conservation, Antigenicity, and Protective Capacity Using Recombinant Protein
Source: PLoS One. 2015 Jun 16;10(6):e0129309. doi: 10.1371/journal.pone.0129309 (PMC4469585; doi:10.1371/journal.pone.0129309)
Supplement: S3 Fig — AMF_640 is the Florida strain homolog of AM854. ACIS_00486 is the A. marginale ss. centrale ortholog of AM854. (DOCX) [file pone.0129309.s003.docx]

AM854_6DE 1 MLHRWLALCFLASFAVTGCGLFSKEKVGMDIVGVPFSAGRVEKVYFDFNKYEIKGSGKKV
AM854_Dawn 1 MLHRWLALCFLASFAVTGCGLFSKEKVGMDIVGVPFSAGRVEKVYFDFNKYEIKGSGKKV
AM854_C51 1 MLHRWLALCFLASFAVTGCGLFSKEKVGMDIVGVPFSAGRVEKVYFDFNKYEIKGSGKKV
Am854_C52 1 MLHRWLALCFLASFAVTGCGLFSKEKVGMDIVGVPFSAGRVEKVYFDFNKYEIKGSGKKV
AM854_EMΦ 1 MLHRWLALCFLASFAVTGCGLFSKEKVGMDIVGVPFSAGRVEKVYFDFNKYEIKGSGKKV
AM854_N3574 1 MLHRWLALCFLASFAVTGCGLFSKEKVGMDIVGVPFSAGRVEKVYFDFNKYEIKGSGKKV
AM854_N4506 1 MLHRWLALCFLASFAVTGCGLFSKEKVGMDIVGVPFSAGRVEKVYFDFNKYEIKGSGKKV
AM854_PR 1 MLHRWLALCFLASFAVTGCGLFSKEKVGMDIVGVPFSAGRVEKVYFDFNKYEIKGSGKKV
AM854_VA 1 MLHRWLALCFLASFAVTGCGLFSKEKVGMDIVGVPFSAGRVEKVYFDFNKYEIKGSGKKV
AM854_StM 1 MLHRWLALCFLASFAVTGCGLFSKEKVGMDIVGVPFSAGRVEKVYFDFNKYEIKGSGKKV
AMF_640 1 MLHRWLALCFLASFAVTGCGLFSKEKVGMDIVGVPFSAGRVEKVYFDFNKYEIKGSGKKV
ACIS_00486 1 MLHRWLALCLLASLAVTGCELFNKEKVNIDIGGVPLSAGRVEKVYFDFNKYEIKGSGKKV


AM854_6DE 61 LLGLVERMKADKRSTLLIIGHTDSRGTEEYNLALGERRANAVKEFILGCDRSLSPRISTQ
AM854_Dawn 61 LLGLVERMKADKRSTLLIIGHTDSRGTEEYNLALGERRANAVKEFILGCDRSLSPRISTQ
AM854_C51 61 LLGLVERMKADKRSTLLIIGHTDSRGTEEYNLALGERRANAVKEFILGCDRSLSPRISTQ
Am854_C52 61 LLGLVERMKADKRSTLLIIGHTDSRGTEEYNLALGERRANAVKEFILGCDRSLSPRISTQ
AM854_EMΦ 61 LLGLVERMKADKRSTLLIIGHTDSRGTEEYNLALGERRANAVKEFILGCDRSLSPRISTQ
AM854_N3574 61 LLGLVERMKADKRSTLLIIGHTDSRGTEEYNLALGERRANAVKEFILGCDRSLSPRISTQ
AM854_N4506 61 LLGLVERMKADKRSTLLIIGHTDSRGTEEYNLALGERRANAVKEFILGCDRSLSPRISTQ
AM854_PR 61 LLGLVERMKADKRSTLLIIGHTDSRGTEEYNLALGERRANAVKEFILGCDRSLSPRISTQ
AM854_VA 61 LLGLVERMKADKRSTLLIIGHTDSRGTEEYNLALGERRANAVKEFILGCDRSLSPRISTQ
AM854_StM 61 LLGLVERMKADKRSTLLIIGHTDSRGTEEYNLALGERRANAVKEFILGCDRSLSPRISTQ
AMF_640 61 LLGLVERMKADKRSTLLIIGHTDSRGTEEYNLALGERRANAVKEFILGCDRSLSPRISTQ
ACIS_00486 61 LLGLVERMKADKMSTLLIVGHTDSRGTEEYNLALGERRANAVKEFILGCDRSLSPRISTQ


AM854_6DE 121 SRGKAEPEVLVYSSDFKEAEKAHAQNRRVVLIVECQHSVSPKKKMAIKWPFSFGRSAAKQ
AM854_Dawn 121 SRGKAEPEVLVYSSDFKEAEKAHAQNRRVVLIVECQHSVSPKKKMAIKWPFSFGRSAAKQ
AM854_C51 121 SRGKAEPEVLVYSSDFKEAEKAHAQNRRVVLIVECQHSVSPKKKMAIKWPFSFGRSAAKQ
Am854_C52 121 SRGKAEPEVLVYSSDFKEAEKAHAQNRRVVLIVECQHSVSPKKKMAIKWPFSFGRSAAKQ
AM854_EMΦ 121 SRGKAEPEVLVYSSDFKEAEKAHAQNRRVVLIVECQHSVSPKKKMAIKWPFSFGRSAAKQ
AM854_N3574 121 SRGKAEPEVLVYSSDFKEAEKAHAQNRRVVLIVECQHSVSPKKKMAIKWPFSFGRSAAKQ
AM854_N4506 121 SRGKAEPEVLVYSSDFKEAEKAHAQNRRVVLIVECQHSVSPKKKMAIKWPFSFGRSAAKQ
AM854_PR 121 SRGKAEPEVLVYSSDFKEAEKAHAQNRRVVLIVECQHSVSPKKKMAIKWPFSFGRSAAKQ
AM854_VA 121 SRGKAEPEVLVYSSDFKEAEKAHAQNRRVVLIVECQHSVSPKKKMAIKWPFSFGRSAAKQ
AM854_StM 121 SRGKAEPEVLVYSSDFKEAEKAHAQNRRVVLIVECQHSVSPKKKMAIKWPFSFGRSAAKQ
AMF_640 121 SRGKAEPEVLVYSSDFKEAEKAHAQNRRVVLIVECQHSVSPKKKMAIKWPFSFGRSAAKQ
ACIS_00486 121 SRGKAEPEILVYSSDFKEAEKAHAQNRRVVLIMECQHAASPKKARVSRWPFSFGRSSATQ


AM854_6DE 181 DDVGSSEVSDENPVDDSSEGIASEEAAPEEGVVSEEAAEEAPEVAQDSSAGVVAPE
AM854_Dawn 181 DDVGSSEVSDENPVDDSSEGIASEEAAPEEGVVSEEAAEEAPEVAQDSSAGVVAPE
AM854_C51 181 DDVGSSEVSDENPVDDSSEGIASEEAAPEEGVVSEEAAEEAPEVAQDSSAGVVAPE
Am854_C52 181 DDVGSSEVSDENPVDDSSEGIASEEAAPEEGVVSEEAAEEAPEVAQDSSAGVVAPE
AM854_EMΦ 181 DDVGSSEVSDENPVDDSSEGIASEEAAPEEGVVSEEAAEEAPEVAQDSPAGVVAPE
AM854_N3574 181 DDVGSSEVSDENPVDDSSEGIASEEAAPEEGVVSEEAAEEAPEVAQDSSAGVVAPE
AM854_N4506 181 DDVGSSEVSDENPVDDSSEGIASEEAAPEEGVVSEEAAEEAPEVAQDSSAGVVAPE
AM854_PR 181 DDVGSSEVSDENPVDDSSEGIASEEAAPEEGVVSEEAAEEAPEVAQDSSAGVVAPE
AM854_VA 181 DDVGSSEVSDENPVDDSSEGIASEEAAPEEGVVSEEAAEEAPEVAQDSSAGVVAPE
AM854_StM 181 DDVGSSEVSDENPVDDSSEGIASEEAAPEEGVVSEEAAEEAPEVAQDSSAGVVAPE
AMF_640 181 DDVGSSEVSDENPVDDSSEGIASEEAAPEEGVVSEEAAEEAPEVAQDSSAGVVAPE
ACIS_00486 181 QDNGGGTVAAGSPGED----------APAEVVEPEETQE--------------AGE


Fig. S3. Amino acid alignment of AM854 for all *A. marginale* strains and isolates and *A. marginale* ss. *centrale*. AMF_640 is the Florida strain homolog of AM854. ACIS_00486 is the *A. marginale* ss. *centrale* ortholog of AM854.
