# Supplementary material for: Subdominant Outer Membrane Antigens in Anaplasma marginale: Conservation, Antigenicity, and Protective Capacity Using Recombinant Protein
Source: PLoS One. 2015 Jun 16;10(6):e0129309. doi: 10.1371/journal.pone.0129309 (PMC4469585; doi:10.1371/journal.pone.0129309)
Supplement: S4 Fig — AMF_717 is the Florida strain homolog of AM936. ACIS_00403 is the A. marginale ss. centrale ortholog of AM936. (DOCX) [file pone.0129309.s004.docx]

AM936_6DE 1 MSGEDEYKEIIRQCIGSVKEVFGEGRFDDVVASIMKMQEKVLASSMKDGDPVGQIAADGV
AM936_Dawn 1 MSGEDEYKEIIRQCIGSVKEVFGEGRFDDVVASIMKMQEKVLASSMKDGDPVGQIAADGV
AM936_C51 1 MSGEDEYKEIIRQCIGSVKEVFGEGRFDDVVASIMKMQEKVLASSMKDGDPVGQIAADGV
AM936_C52 1 MSGEDEYKEIIRQCIGSVKEVFGEGRFDDVVASIMKMQEKVLASSMKDGDPVGQIAADGV
AM936_EMΦ 1 MSGEDEYKEIIRQCIGSVKEVFGEGRFDDVVASIMKMQEKVLASSMKDGDPVGQIAADGV
AM936_N3518 1 MSGEDEYKEIIRQCIGSVKEVFGEGRFDDVVASIMKMQEKVLASSMKDGDPVGQIAADGV
AM936_N4506 1 MSGEDEYKEIIRQCIGSVKEVFGEGRFDDVVASIMKMQEKVLASSMKDGDPVGQIAADGV
AM936_PR 1 MSGEDEYKEIIRQCIGSVKEVFGEGRFDDVVASIMKMQEKVLASSMKDGDPVGQIAADGV
AM936_VA 1 MSGEDEYKEIIRQCIGSVKEVFGEGRFDDVVASIMKMQEKVLASSMKDGDPVGQIAADGV
AM936_StM 1 MSGEDEYKEIIRQCIGSVKEVFGEGRFDDVVASIMKMQEKVLASSMKDGDPVGQIAADGV
AMF_717 1 MSGEDEYKEIIRQCIGSVKEVFGEGRFDDVVASIMKMQEKVLASSMKDGDPVGQIAADGV
ACIS_00403 1 MSGEDEYKEIIRQCIGSVKEVFGEGRFDDVVASIMKMQEKVLASSMKDGDPVGQIAADGV


AM936_6DE 61 GNELYDRIADRLEERVSQKISEDLRIIKKRLLRLERVVLGGGSVSGDAAA-HQVSGNQPS
AM936_Dawn 61 GNELYDRIADRLEERVSQKISEDLRIIKKRLLRLERVVLGGGSVSGDAAA-HQVSGNQPS
AM936_C51 61 GNELYDRIADRLEERVSQKISEDLRIIKKRLLRLERVVLGGGSVSGDAAA-HQVSGNQPS
AM936_C52 61 GNELYDRIADRLEERVSQKISEDLRIIKKRLLRLERVVLGGGSVSGDAAA-HQVSGNQPS
AM936_EMΦ 61 GNELYDRIADRLEERVSQKISEDLRIIKKRLLRLERVVLGGGSVSGDAAA-HQVSGNQPS
AM936_N3518 61 GNELYDRIADRLEERVSQKISEDLRIIKKRLLRLERVVLGGGSVSGDAAA-HQVSGNQPS
AM936_N4506 61 GNELYDRIADRLEERVSQKISEDLRIIKKRLLRLERVVLGGGSVSGDAAA-HQVSGNQPS
AM936_PR 61 GNELYDRIADRLEERVSQKISEDLRIIKKRLLRLERVVLGGGSVSGDAAA-HQVSGNQPS
AM936_VA 61 GNELYDRIADRLEERVSQKISEDLRIIKKRLLRLERVVLGGGSVSGDAAA-HQVSGNQPS
AM936_StM 61 GNELYDRIADRLEERVSQKISEDLRIIKKRLLRLERVVLGGGSVSGDAAA-HQVSGNQPS
AMF_717 61 GNELYDRIADRLEERVSQKISEDLRIIKKRLLRLERVVLGGGSVSGDAAA-HQVSGNQPS
ACIS_00403 61 GNELYDRIADRLEERVSQKISEDLRIIKKRLLRLERVVLGGGSVSGDAAAAHQVSGNQPS


AM936_6DE 120 QQNSSAAAEGG
AM936_Dawn 120 QQNSSAAAEGG
AM936_C51 120 QQNSSAAAEGG
AM936_C52 120 QQNSSAAAEGG
AM936_EMΦ 120 QQNSSAAAEGG
AM936_N3518 120 QQNSSAAAEGG
AM936_N4506 120 QQNSSAAAEGG
AM936_PR 120 QQNSSAAAEGG
AM936_VA 120 QQNSSAAAEGG
AM936_StM 120 QQNSSAAAEGG
AMF_717 120 QQNSSAAAEGG
ACIS_00403 121 QQNSSAAAEGG

Fig. S4. Amino acid alignment of AM936 for all *A. marginale* strains and isolates. AMF_717 is the Florida strain homolog of AM936. ACIS_00403 is the *A. marginale* ss. *centrale* ortholog of AM936.
