# Supplementary material for: Subdominant Outer Membrane Antigens in Anaplasma marginale: Conservation, Antigenicity, and Protective Capacity Using Recombinant Protein
Source: PLoS One. 2015 Jun 16;10(6):e0129309. doi: 10.1371/journal.pone.0129309 (PMC4469585; doi:10.1371/journal.pone.0129309)
Supplement: S5 Fig — N3518.1 and N3518.2 are multiple variants that were obtained from the same isolate. VA.1 and VA.2 are multiple variants that were obtained from the Virginia strain. AMF_790 is the Florida strain homolog of AM1041. ACIS_00314 is the A. marginale ss. centrale ortholog of AM1041. (DOCX) [file pone.0129309.s005.docx]

AM1041_6DE 1 MTSLSTKHAHHIAVANTAHAVSGVLISMGILLASLSILAVFLAVLMYFSAAARIFSLSAT
AM1041_Dawn 1 MTSLSTKHAHHIAVANTAHAVSGVLISMGILLASLSILAVFLAVLMYFSAAARIFSLSAT
AM1041_C51 1 MTSLSTKHAHHIAVANTAHAVSGVLISMGILLASLSILAVFLAVLMYFSAAARIFSLSAT
AM1041_C52 1 MTSLSTKHAHHIAVANTAHAVSGVLISMGILLASLSILAVFLAVLMYFSAAARIFSLSAT
AM1041_EMΦ 1 MTSLSTKHAHHIAVANTAHAVSGVLISMGILLASLSILAVFLAVLMYFSAAARIFSLSAT
AM1041_N3518.1 1 MTSLSTKHAHHIAVANTAHAVSGVLISMGILLASLSILAVFLAVLMYFSAAARIFSLSAT
AM1041_N3518.2 1 MTSLSTKHAHHIAVANTAHAVSGVLISMGILLASLSILAVFLAVLMYFSAAARIFSLSAT
AM1041_N3571 1 MTSLSTKHAHHIAVANTAHAVSGVLISMGILLASLSILAVFLAVLMYFSAAARIFSLSAT
AM1041_PR 1 MTSLSTKHAHHIAVANTAHAVSGVLISMGILLASLSILAVFLAVLMYFSAAARIFSLSAT
AM1041_VA.1 1 MTSLSTKHAHHIAVANTAHAVSGVLISMGILLASLSILAVFLAVLMYFSAAARIFSLSAT
AM1041_VA.2 1 MTSLSTKHAHHIAVANTAHAVSGVLISMGILLASLSILAVFLAVLMYFSAAARIFSLSAT
AM1041_StM 1 MTSLSTKHAHHIAVANTAHAVSGVLISMGILLASLSILAVFLAVLMYFSAAARIFSLSAT
AMF_790 1 MTSLSTKHAHHIAVANTAHAVSGVLISMGILLASLSILAVFLAVLMYFSAAARIFSLSAT
ACIS_00314 1 MTSLSTKHAHHIAVANTAHAVSGVLISMGILLASLSILAVFLAVLMYFSAAARIFSLSAT


AM1041_6DE 61 NAATVCAAVALLSACIVFCSVRDLVKSCKQVKQAGKEESPAVQPLQPILTSVTPHSEAAA
AM1041_Dawn 61 NAATVCAAVALLSACIVFCSVRDLVKSCKQVKQAGKEESPAVQPLQPILTSVTPHSEAAA
AM1041_C51 61 NAATVCAAVALLSACIVFCSVRDLVKSCKQVKQAGKEESPAVQPLQPILTSVTPHSEAAA
AM1041_C52 61 NAATVCAAVALLSACIVFCSVRDLVKSCKQVKQAGKEESPAVQPLQPILTSVTPHSEAAA
AM1041_EMΦ 61 NAATVCAAVALLSACIVFCSVRDLVKSCKQVKQAGKEESPAVQPLQPILTSVTPHSEAAA
AM1041_N3518.1 61 NAATVCAAVALLSACIVFCSVRDLVKSCKQVKQAGKEESPAVQPLQPILTSVTPHSEAAA
AM1041_N3518.2 61 NAATVCAAVALLSACIVFCSVRDLVKSCKQVKQAGKEESPAVQPLQPILTSVTPHSEAAA
AM1041_N3571 61 NAATVCAAVALLSACIVFCSVRDLVKSCKQVKQAGKEESPAVQPLQPILTSVTPHSEAAA
AM1041_PR 61 NAATVCAAVALLSACIVFCSVRDLVKSCKQVKQAGKEESPAVQPLQPILTSVTPHSEAAA
AM1041_VA_1 61 NAATVCAAVALLSACIVFCSVRDLVKSCKQVKQAGKEESPAVQPLQPILTSVTPHSEAAA
AM1041_VA_2 61 NAATVCAAVALLSACIVFCSVRDLVKSCKQVKQAGKEESPAVQPLQPILTSVTPHSEAAA
AM1041_StM 61 NAATVCAAVALLSACIVFCSVRDLVKSCKQVKQAGKEESPAVQPLQPILTSVTPHSEAAA
AMF_790 61 NAATVCAAVALLSACIVFCSVRDLVKSCKQVKQAGKEESPAVQPLQPILTSVTPHSEAAA
ACIS_00314 61 NAATVCAAVALLSACIVFCSVRDLVKSCKQVKQAGKEESPAVQPLQPILTSVTPHSEAAA


AM1041_6DE 121 NTRRQQRIRGSKHGDVIAEIANSVASLAMIATFTALATVVVVAIARIFNSSVAFIGAYVY
AM1041_Dawn 121 NTRRQQRIRGSKHGDVIAEIANSVASLAMIATFTALATVVVVAIARIFNSSVV-IGAYVY
AM1041_C51 121 NTRRQQRIRGSKHGDVIAEIANSVASLAMIATFTALATVVVVAIARIFNSSVV-IGAYVY
AM1041_C52 121 NTRRQQRIRGSKHGDVIAEIANSVASLAMIATFTALATVVVVAIARIFNSSVAFIGAYVY
AM1041_EMΦ 121 NTRRQQRIRGSKHGDVIAEIANSVASLAMIATFTALATVVVVAIASIFNSSVAFIGAYVY
AM1041_N3518.1 121 NTRRQQRIRGSKHGDVIAEIANSVASLAMIATFTALATVVVVAIARIFNSSVV-IGAYVY
AM1041_N3518.2 121 NTRRQQRIRGSKHGDVIAEIANSVASLAMIATFTALATVVVVAIARIFNSSVV-IGAYVY
AM1041_N3571 121 NTRRQQRIRGSKHGDVIAEIANSVASLAMIATFTALATVVVVAIARIFNSSVV-IGAYVY
AM1041_PR 121 NTRRQQRIRGSKHGDVIAEIANSVASLAMIATFTALATVVVVAIARIFNSSVAFIGAYVY
AM1041_VA_1 121 NTRRQQRIRGSKHGDVIAEIANSVASLAMIATFTALATVVVVAIARIFNSSVAFIGAYVY
AM1041_VA_2 121 NTRRQQRIRGSKHGDVIAEIANSVASLAMIATFTALATVVVVAIARIFNSSVV-IGAYVY
AM1041_StM 121 NTRRQQRIRGSKHGDVIAEIANSVASLAMIATFTALATVVVVAIARIFNSSVV-IGAYVY
AMF_790 121 NTRRQQRIRGSKHGDVIAEIANSVASLAMIATFTALATVVVVAIARIFNSSVAFIGAYVY
ACIS_00314 121 NTRRQQRIRGSKHGDVIAEIANSVASLAMIATFTALATVVGVAIARIFNSSVAFVGADVY


AM1041_6DE 181 STDAFGTAKVAIVAAAAAVISAVVTALFAAIAARAANRSTMPAIPPEDKTATDKAVTQVK
AM1041_Dawn 180 GTDAFGTAKVAIVAAAAAVISAVVTALFAAIAARAANRSTMPAIPPEDKTATDKAVTQVK
AM1041_C51 180 GTDAFGTAKVAIVAAAAAVISAVVTALFAAIAARAANRSTMPAIPPEDKTATDKAVTQVK
AM1041_C52 181 STDAFGTAKVAIVAAAVAVISAVVTALFAAIAARAANRSTMPAIPPEDKTATDKAVTQVK
AM1041_EMΦ 181 STDAFGTAKVAIVAAAAAVISAVVTALFAAIAARAANRSTMPAIPPEDKTATNKAVTQVK
AM1041_N3518.1 180 GTDAFGTAKVAIVAAAAAVISAVVTALFAAIAARAANRSTMPAIPPEDKTATDKAVTQVK
AM1041_N3518.2 180 GTDAFGTAKVAIVAAAAAVISAVVTALFAAIAARAANRSTMPAIPPEDKTATDKAVTQVK
AM1041_N3571 180 GTDAFGTAKVAIVAAAAAVISAVVTALFAAIAARAANRSTMPAIPPEDKTATDKAVTQVK
AM1041_PR 181 STDAFGTAKVAIVAAAAAVISAVVTALFAAIAARAANRSTMPAIPPEDKTATDKAVTQVK
AM1041_VA_1 181 STDAFGTAKVAIVAAAAAVISAVVTALFAAIAARAANRSTMPAIPPEDKTATDKAVTQVK
AM1041_VA_2 180 GTDAFGTAKVAIVAAAAAVISAVVTALFAAIAARAANRSTMPAIPPEDKTATDKAVTQVK
AM1041_StM 180 GTDAFGTAKVAIVAAAAAVISAVVTALFAAIAARAANRSTMPAIPPEDKTATDKAVTQVK
AMF_790 181 STDAFGTAKVAIVAAAAAVISAVVTALFAAIAARAANRSTMPAIPPEDKTATDKAVTQVK
ACIS_00314 181 STDAFGTAKVAIVAAAAAVISAVVTALFAAIAARAANRSTMPAIPPEDKTATDKAVTQVK
AM1041_6DE 241 HANLAIKISGITAIAATSTAAVAALIVPFAAKASEAFAISWGMTTAARTAAATTHTTGII
AM1041_Dawn 240 HANLAIKISGITAIAATSTAAVAALIVPFAAKASEAFAISWGMTTAARTAAATTHTTGIV
AM1041_C51 240 HANLAIKISGITAIAATSTAAVAALIVPFAAKASEAFAISWGMTTAARTAAATTHTTGII
AM1041_C52 241 HANLAIKISGITAIAATSTAAVAALIVPFAAKASEAFAISWGMTTAARTAAATTHTTGIV
AM1041_EMΦ 241 HANLAIKISGITAIAATSTAAVAALIVPFAAKASEAFAISWGMTTAARTAAATTHTTGII
AM1041_N3518.1 240 HANLAIKISGITAIAATSTAAVAALIVPFAAKASEAFAISWGMTTAARTAAATTHTTGII
AM1041_N3518.2 240 HANLAIKISGITAIAATSTAAVAALIVPFAAKASEAFAISWGMTTAARTAAATTHTTGII
AM1041_N3571 240 HANLAIKISGITAIAATSTAAVAALIVPFAAKASEAFAISWGMTTAARTAAATTHTTGII
AM1041_PR 241 HANLAIKISGITAIAATSTAAVAALIVPFAAKASEAFAISWGMTTAARTAAATTHTTGII
AM1041_VA_1 241 HANLAIKISGITAIAATSTAAVAALIVPFAAKASEAFAISWGMTTAARTAAATTHTTGII
AM1041_VA_2 240 HANLAIKISGITAIAATSTAAVAALIVPFAAKASEAFAISWGMTTAARTAAATTHTTGIV
AM1041_StM 240 HANLAIKISGITAIAATSTAAVAALIVPFAAKASEAFAISWGMTTAARTAAATTHTTGII
AMF_790 241 HANLAIKISGITAIAATSTAAVAALIVPFAAKASEAFAISWGMTTAARTAAATTHTTGII
ACIS_00314 241 HANLAIKISGITAIAATSTAAVAALIVPFAAKASEAFAISWGMTTAARTAAATTHTTGII


AM1041_6DE 301 GTVSTAAYLPHALIALSVACSLCLLIHIAVDIYLARIPSSQPRGKQRISHQVADRARSTF
AM1041_Dawn 300 GSVSTAAYLPHALIALSVACSLCLLIHIAVDIYLARIPSSQPRGKQRISHQVADRARSTF
AM1041_C51 300 GTVSTAAYLPHALIALSVACSLCLLIHIAVDIYLARIPSSQPRGKQRISHQVADRARSTF
AM1041_C52 301 GSVSTAAYLPHALIALSVACSLCLLIHIAVDIYLARIPSSQPRGKQRISHQVADRARSTF
AM1041_EMΦ 301 GTVSTAAYLPHALIALSVACSLCLLIHIAVDIYLARIPSSQPRGKQRISHQVADRARSTF
AM1041_N3518.1 300 GTVSTAAYLPHALIALSVACSLCLLIHIAVDIYLARIPSSQPRGKQRISHQVADRARSTF
AM1041_N3518.2 300 GTVSTAAYLPHALIALSVACSLCLLIHIAVDIYLARIPSSQPRGKQRISHQVADRARSTF
AM1041_N3571 300 GTVSTAAYLPHALIALSVACSLCLLIHIAVDIYLARIPSSQPRGKQRISHQVADRARSTF
AM1041_PR 301 GTVSTAAYLPHALIALSVACSLCLLIHIAVDIYLARIPSSQPRGKQRISHQVADRARSTF
AM1041_VA_1 301 GTVSTAAYLPHALIALSVACSLCLLIHIAVDIYLARIPSSQPRGKQRISHQVADRARSTF
AM1041_VA_2 300 GSVSTAAYLPHALIALSVACSLCLLIHIAVDIYLARIPSSQPRGKQRISHQVADRARSTF
AM1041_StM 300 GTVSTAAYLPHALIALSVACSLCLLIHIAVDIYLARIPSSQPRGKQRISHQVADRARSTF
AMF_790 301 GTVSTAAYLPHALIALSVACSLCLLIHIAVDIYLARIPSSQPRGKQRISHQVADRARSTF
ACIS_00314 301 GTVSTAAYLPHALIALSVACSLCLLIHIAVDIYLARIPSSQPRGKQRISHQVADRARSTF


AM1041_6DE 361 SKGNSLQPKSAPASPHDDPTRGHSDFKHNNPADRNMTSRPCTPPTAADPKHTTLVAAISA
AM1041_Dawn 360 SKGNSLQPKSAPASPHDDPTRGHSDFKHNNPAGR---SMPCTSPTAADPKHTTLVAAISV
AM1041_C51 360 SKGNSLQPKSAPASPHDDPTRGHSDFKHNNPADRNITSRPCTPPTAADPKHTTLVAAISA
AM1041_C52 361 SKGNSLQPKSAPASPHDDPTRGHSDFKHNNPADRNITSRPCTPPTAADPKHTTLVAAISA
AM1041_EMΦ 361 SKGNSLQPKSAPASPHDDPTRGHSDFKHNNPADRNMTSRPCTPPTAADPKHTTLVAAISA
AM1041_N3518.1 360 SKGNSLQPKSAPASPHDDPTRGHSDFKHNNPAGRNITSMPCTSPTAADPKHTTLVAAISV
AM1041_N3518.2 360 SKGNSLQPKSAPASPHDDPTRGHSDFKHNNPAGR---SMPCTSPTAADPKHTTLVAAISV
AM1041_N3571 360 SKGNSLQPKSAPASPHDYPTRGHSAFKHNNPAGR---SMPCTSPTAADPKHTTLVAAISV
AM1041_PR 361 SKGNSLQPKSAPASPHDDPTRGHSDFKHNNPADRNMTSRPCTPPTAADPKHTTLVAAISA
AM1041_VA_1 361 SKGNSLQPKSAPASPHDDPTRGHSDFKHNNPADRNMTSRPCTPPTAADPKHTTLVAAISA
AM1041_VA_2 360 SKGNSLQPKSAPASPHDDPTRGHSDFKHNNPADRNMTSRPCTPPTAADPKHTTLVAAISA
AM1041_StM 360 SKGNSLQPKSAPASPHDDPTRGHSDFKHNNPADRNITSRPCTPPTAADPKHTTLVAAISA
AMF_790 361 SKGNSLQPKSAPASPHDDPTRGHSDFKHNNPADRNMTSRPCTPPTAADPKHTTLVAAISA
ACIS_00314 361 SKGNSLQPKSAPASPHDDPTRGHSDFKHNNPADRNMTSRPCTPPTAADPKHTTLVAAISA


AM1041_6DE 421 VMICSAAFLMRNYAMLGAVAPPAALLLYGVIAWVRKMSCHEVQEEVLSSKIDSPTAAMLP
AM1041_Dawn 417 VMICSAAFLMRNYAMLGAVSPAAALLLYGVIAWVRKMSCHEVQEEVLSSKIDSPTAAMLP
AM1041_C51 420 VMICSAAFLMRNYAMLGAVAPPAALLLYGVIAWVRKMSCHEVQEEVLSSKIDSPTAAMLP
AM1041_C52 421 VMICSAAFLMRNYAMLGAVAPPAALLLYGVIAWVRKMSCHEVQEEVLSSKIDSPTAAMLP
AM1041_EMΦ 421 VMICSAAFLMRNYAMLGAVAPPAALLLYGVIAWVRKMSCNEVQEEVLSSKIDGPTAAMLP
AM1041_N3518.1 420 VMICSAAFLMRNYAMLGAVSPAAALLLYGVIAWVRKMSCHEVQEEVLSSKIDSPTAAMLP
AM1041_N3518.2 417 VMICSAAFLMRNYAMLGAVSPAAALLLYGVIAWVRKMSCHEVQEEVLSSKIDSPTAAMLP
AM1041_N3571 417 VMICSAAFLMRNYAMLGAVSPAAALLLYGVIAWVRKMSCHEVQEEVLSSKIDSPTAAMLP
AM1041_PR 421 VMICSAAFLMRNYAMLGAVAPPAALLLYGVIAWVRKMSCHEVQEEVLSSKIDSPTAAMLP
AM1041_VA_1 421 VMICSAAFLMRNYAMLGAVAPPAALLLYGVIAWVRKMSCHEVQEEVLSSKIDSPTAAMLP
AM1041_VA_2 420 VMICSAAFLMRNYAMLGAVAPPAALLLYGVIAWVRKMSCNEVQEEVLSSKIDGPTAAMLP
AM1041_StM 420 VMICSAAFLMRNYAMLGAVAPPAALLLYGVIAWVRKMSCHEVQEEVLSSKIDSPTAAMLP
AMF_790 421 VMICSAAFLMRNYAMLGAVAPPAALLLYGVIAWVRKMSCHEVQEEVLSSKIDSPTAAMLP
ACIS_00314 421 VMICSAAFLMRNYAMLGAVAPPAALLLYGVIAWVRKMSCNEVQEEVLSSKIDGPTAAMLP
AM1041_6DE 481 HVYDVALQTGKR
AM1041_Dawn 477 HMYDVALQTGKR
AM1041_C51 480 HVYDVALQTGKR
AM1041_C52 481 HVYDVALQTGKR
AM1041_EMΦ 481 HMYDVALQTGKR
AM1041_N3518.1 480 HMYDVALQTGKR
AM1041_N3518.2 477 HMYDVALQTGKR
AM1041_N3571 477 HMYDVALQTGKR
AM1041_PR 481 HVYDVALQTGKR
AM1041_VA.1 481 HVYDVALQTGKR
AM1041_VA.2 480 HMYDVALQTGN
AM1041_StM 480 HVYDVALQTGKR
AMF_790 481 HVYDVALQTGKR
ACIS_00314 481 HMYDVALQTG


Fig. S5. Amino acid alignment of AM1041 for all *A. marginale* strains and isolates and *A. marginale* ss. *centrale*. N3518.1 and N3518.2 are multiple variants that were obtained from the same isolate. VA.1 and VA.2 are multiple variants that were obtained from the Virginia strain. AMF_790 is the Florida strain homolog of AM1041. ACIS_00314 is the *A. marginale* ss. *centrale* ortholog of AM1041.
