# Supplementary material for: Subdominant Outer Membrane Antigens in Anaplasma marginale: Conservation, Antigenicity, and Protective Capacity Using Recombinant Protein
Source: PLoS One. 2015 Jun 16;10(6):e0129309. doi: 10.1371/journal.pone.0129309 (PMC4469585; doi:10.1371/journal.pone.0129309)
Supplement: S6 Fig — AMF_828 is the Florida strain homolog of AM1096. ACIS_00268 is the A. marginale ss. centrale ortholog of AM1096. (DOCX) [file pone.0129309.s006.docx]

AM1096_6DE 1 MRYVLVFVLYVVACVSFVGPILPASAVAGVTRVKVIGNERLDSGTVQFYAKVPLTGDVTQ
AM1096_Dawn 1 MRYVLVFVLYVVACVSFVGPILPASAVAGVTRVKVIGNERLDSGTVQFYAKVPLTGDVTQ
AM1096_C51 1 MRYVLVFVLYVVACVSFVGPILPASAVAGVTRVKVIGNERLDSGTVQFYAKVPLTGDVTQ
AM1096_C52 1 MRYVLVFVLYVVACVSFVGPILPASAVAGVTRVKVIGNERLDSGTVQFYAKVPLTGDVTQ
AM1096_EMΦ 1 MRYVLVFVLYVVACVSFVGPILPASAVAGVTRVKVIGNERLDSGTVQFYAKVPLTGDVTQ
AM1096_N4506 1 MRYVLVFVLYVVACVSFVGPILPASAVAGVTRVKVIGNERLDSGTVQFYAKVPLTGDVTQ
AM1096_N3571 1 MRYVLVFVLYVVACVSFVGPILPASAVAGVTRVKVIGNERLDSGTVQFYAKVPLTGDVTQ
AM1096_PR 1 MRYVLVFVLYVVACVSFVGPILPASAVAGVTRVKVIGNERLDSGTVQFYAKVPLTGDVTQ
AM1096_VA 1 MRYVLVFVLYVVACVSFVGPILPASAVAGVTRVKVIGNERLDSGTVQFYAKVPLTGDVTQ
AM1096_StM 1 MRYVLVFVLYVVACVSFVGPILPASAVAGVTRVKVIGNERLDSGTVQFYAKVPLTGDVTQ
AMF_828 1 MRYVLVFVLYVVACVSFVGPILPASAVAGVTRVKVIGNERLDSGTVQFYAKVPLTGDVTQ
ACIS_00268 1 MRYIFVFALYVVACVSIVGSVVPASAAAGVTRVRVVGNERLDSGTVQFYAKVPLTGDVTQ


AM1096_6DE 61 PEIDEAIKNLYATSLFSRVSVRAEGNELVIRVKENPVVRSIKIVGNRMFSDKNLESDVLK
AM1096_Dawn 61 PEIDEAIKNLYATSLFSRVSVRAEGNELVIRVKENPVVRSIKIVGNRMFSDKNLESDVLK
AM1096_C51 61 PEIDEAIKNLYATSLFSRVSVRAEGNELVIRVKENPVVRSIKIVGNRMFSDKNLESDVLK
AM1096_C52 61 PEIDEAIKNLYATSLFSRVSVRAEGNELVIRVKENPVVRSIKIVGNRMFSDKNLESDVLK
AM1096_EMΦ 61 PEIDEAIKNLYATSLFSRVSVRAEGNELVIRVKENPVVRSIKIVGNRMFSDKNLESDVLK
AM1096_N4506 61 PEIDEAIKNLYATSLFSRVSVRAEGNELVIRVKENPVVRSIKIVGNRMFSDKNLESDVLK
AM1096_N3571 61 PEIDEAIKNLYATSLFSRVSVRAEGNELVIRVKENPVVRSIKIVGNRMFSDKNLESDVLK
AM1096_PR 61 PEIDEAIKNLYATSLFSRVSVRAEGNELVIRVKENPVVRSIKIVGNRMFSDKNLESDVLK
AM1096_VA 61 PEIDEAIKNLYATSLFSRVSVRAEGNELVIRVKENPVVRSIKIVGNRMFSDKNLESDVLK
AM1096_StM 61 PEIDEAIKNLYATSLFSRVSVRAEGNELVIRVKENPVVRSIKIVGNRMFSDKNLESDVLK
AMF_828 61 PEIDEAIKNLYATSLFSRVSVRAEGNELVIRVKENPVVRSIKIVGNRMFSDKNLESDVLK
ACIS_00268 61 PQIDEAIKNLYSTSLFSRVGVRVEGNELVIRVKENPVVRSIKIVGNRVFSDKNLENDVLK


AM1096_6DE 121 MKKMSIFTEAKLKRDLSTLHALYQSRGMLGAKVSYAVRRAPHNAVDIVLNIVEGKATRIG
AM1096_Dawn 121 MKKMSIFTEAKLKRDLSTLHALYQSRGMLGAKVSYAVRRAPHNAVDIVLNIVEGKATRIG
AM1096_C51 121 MKKMSIFTEAKLKRDLSTLHALYQSRGMLGAKVSYAVRRAPHNAVDIVLNIVEGKATRIG
AM1096_C52 121 MKKMSIFTEAKLKRDLSTLHALYQSRGMLGAKVSYAVRRAPHNAVDIVLNIVEGKATRIG
AM1096_EMΦ 121 MKKMSIFTEAKLKRDLSTLHALYQSRGMLGAKVSYAVRRAPHNAVDIVLNIVEGKATRIG
AM1096_N4506 121 MKKMSIFTEAKLKRDLSTLHALYQSRGMLGAKVSYAVRRAPHNAVDIVLNIVEGKATRIG
AM1096_N3571 121 MKKMSIFTEAKLKRDLSTLHALYQSRGMLGAKVSYAVRRAPHNAVDIVLNIVEGKATRIG
AM1096_PR 121 MKKMSIFTEAKLKRDLSTLHALYQSRGMLGAKVSYAVRRAPHNAVDIVLNIVEGKATRIG
AM1096_VA 121 MKKMSIFTEAKLKRDLSTLHALYQSRGMLGAKVSYAVRRAPHNAVDIVLNIVEGKATRIG
AM1096_StM 121 MKKMSIFTEAKLKRDLSTLHALYQSRGMLGAKVSYAVRRAPHNAVDIVLNIVEGKATRIG
AMF_828 121 MKKMSIFTEAKLKRDLSTLHALYQSRGMLGAKVSYAVRRAPHNAVDIVLNIVEGKATRIG
ACIS_00268 121 MKKMSIFTEAKLKRDLSTLHALYQSRGMLGAKVSYAVRRAPHNAVDIVLKIVEGRSTRIG


AM1096_6DE 181 EIRFVGNKAFSSSELKAIVYSKEYKLKEGFGLFGGSTKFFAERLVADQSILREFYTSRGF
AM1096_Dawn 181 EIRFVGNKAFSSSELKAIVYSKEYKLKEGFGLFGSSTKFFAERLVADQSILREFYTSRGF
AM1096_C51 181 EIRFVGNKAFSSSELKAIVYSKEYKLKEGFGLFGGSTKFFAERLVADQSILREFYTSRGF
AM1096_C52 181 EIRFVGNKAFSSSELKAIVYSKEYKLKEGFGLFGGSTKFFAERLVADQSILREFYTSRGF
AM1096_EMΦ 181 EIRFVGNKAFSSSELKAIVYSKEYKLKEGFGLFGGSTKFFAERLVADQSILREFYTSRGF
AM1096_N4506 181 EIRFVGNKAFSSSELKAIVYSKEYKLKEGFGLFGGSTKFFAERLVADQSILREFYTSRGF
AM1096_N3571 181 EIRFVGNKAFSSSELKAIVYSKEYKLKEGFGLFGSSTKFFAERLVADQSILREFYTSRGF
AM1096_PR 181 EIRFVGNKAFSSSELKAIVYSKEYKLKEGFGLFGGSTKFFAERLVADQSILREFYTSRGF
AM1096_VA 181 EIRFVGNKAFSSSELKAIVYSKEYKLKEGFGLFGGSTKFFAERLVADQSILREFYTSRGF
AM1096_StM 181 EIRFVGNKAFSSSELKAIVYSKEYKLKEGFGLFGGSTKFFAERLVADQSILREFYTSRGF
AMF_828 181 EIRFVGNKAFSSSELKAIVYSKEYKLKEGFGLFGGSTKFFAERLVADQSILREFYTSRGF
ACIS_00268 181 EIRFVGNREFPSSKLKAVIYSREHKLKEAFGLLGSSTKFLSERLMIDQSLLREFYTSRGF

AM1096_6DE 241 LDFRVKSVVSEVSRDLTRMTVIFSVEEGKKYQFGESTVTVSDIAHSYEGIEKDLLELVLS
AM1096_Dawn 241 LDFRVKSVVSEVSRDLTRMTVIFSVEEGKKYQFGESTVTVSDIAHSYEGIEKDLLELVLS
AM1096_C51 241 LDFRVKSVVSEVSRDLTRMTVIFSVEEGKKYQFGESTVTVSDIAHSYEGIEKDLLELVLS
AM1096_C52 241 LDFRVKSVVSEVSRDLTRMTVIFSVEEGKKYQFGESTVTVSDIAHSYEGIEKDLLELVLS
AM1096_EMΦ 241 LDFRVKSVVSEVSRDLTRMTVIFSVEEGKKYQFGESTVTVSDIAHSYEGIEKDLLELVLS
AM1096_N4506 241 LDFRVKSVVSEVSRDLTRMTVIFSVEEGKKYQFGESTVTVSDIAHSYEGIEKDLLELVLS
AM1096_N3571 241 LDFRVKSVVSEVSRDLTRMTVIFSVEEGKKYQFGESTVTVSDIAHSYEGIEKDLLELVLS
AM1096_PR 241 LDFRVKSVVSEVSRDLTRMTVIFSVEEGKKYQFGESTVTVSDIAHSYEGIEKDLLELVLS
AM1096_VA 241 LDFRVKSVVSEVSRDLTRMTVIFSVEEGKKYQFGESTVTVSDIAHSYEGIEKDLLELVLS
AM1096_StM 241 LDFRVKSVVSEVSRDLTRMTVIFSVEEGKKYQFGESTVTVSDIAHSYEGIEKDLLELVLS
AMF_828 241 LDFRVKSVVSEVSRDLTRMTVIFSVEEGKKYQFGESTVTVSDIAHSYEGIEKDLLELVLS
ACIS_00268 241 LDFRVKSVVSEVSRDLTRATVVFSVEEGKKYQFGDSIVTVRDVAHSYEGIEEDLLGLVLS

AM1096_6DE 301 KKGEVFDATMVNGSVARMTAHLNERGNLFASVKSDYDVQGDVVNVKYSISLGHSVYIHRI
AM1096_Dawn 301 KKGEVFDATMVNGSVARMTAHLNERGNLFASVKSDYDVQGDVVNVKYSISLGHSVYIHRI
AM1096_C51 301 KKGEVFDATMVNGSVARMTAHLNERGNLFASVKSDYDVQGDVVNVKYSISLGHSVYIHRI
AM1096_C52 301 KKGEVFDATMVNGSVARMTAHLNERGNLFASVKSDYDVQGDVVNVKYSISLGHSVYIHRI
AM1096_EMΦ 301 KKGEVFDATMVNGSVARMTAHLNERGNLFASVKSDYDVQGDVVNVKYSISLGHSVYIHRI
AM1096_N4506 301 KKGEVFDATMVNGSVARMTAHLNERGNLFASVKSDYDVQGDVVNVKYSISLGHSVYIHRI
AM1096_N3571 301 KKGEVFDATMVNGSVARMTAHLNERGNLFASVKSDYDVQGDVVNVKYSISLGHSVYIHRI
AM1096_PR 301 KKGEVFDATMVNGSVARMTAHLNERGNLFASVKSDYDVQGDVVNVKYSISLGHSVYIHRI
AM1096_VA 301 KKGEVFDATMVNGSVARMTAHLNERGNLFASVKSDYDVQGDVVNVKYSISLGHSVYIHRI
AM1096_StM 301 KKGEVFDATMVNGSVARMTAHLNERGNLFASVKSDYDVQGDVVNVKYSISLGHSVYIHRI
AMF_828 301 KKGEVFDATMVNGSVARMTAHLNERGNLFASVKSDYDVQGDVVNVKYSISLGHSVYIHRI
ACIS_00268 301 KKGAVFDATMVNGSVASMTAHLNERGNLFASVTSDYDVQGDVVNVKYSVSLGHSVYIHRI


AM1096_6DE 361 NILGNNRTLDHVMRRKLGIHEGDVYSTGAVRQSRKRLADMDFFETVDVETQKISDSLVDL
AM1096_Dawn 361 NILGNNRTLDHVMRRKLGIHEGDVYSTGAVRQSRKRLADMDFFETVDVETQKISDSLVDL
AM1096_C51 361 NILGNNRTLDHVMRRKLGIHEGDVYSTGAVRQSRKRLADMDFFETVDVETQKISDSLVDL
AM1096_C52 361 NILGNNRTLDHVMRRKLGIHEGDVYSTGAVRQSRKRLADMDFFETVDVETQKISDSLVDL
AM1096_EMΦ 361 NILGNNRTLDHVMRRKLGIHEGDVYSAGAVRQSRKRLADMDFFETVDVETQKISDSLVDL
AM1096_N4506 361 NILGNNRTLDHVMRRKLGIHEGDVYSTGAVRQSRKRLADMDFFETVDVETQKISDSLVDL
AM1096_N3571 361 NILGNNRTLDHVMRRKLGIHEGDVYSTGAVRQSRKRLADMDFFETVDVETQKISDSLVDL
AM1096_PR 361 NILGNNRTLDHVMRRKLGIHEGDVYSTGAVRQSRKRLADMDFFETVDVETQKISDSLVDL
AM1096_VA 361 NILGNNRTLDHVMRRKLGIHEGDVYSTGAVRQSRKRLADMDFFETVDVETQKISDSLVDL
AM1096_StM 361 NILGNNRTLDHVMRRKLGIHEGDVYSTGAVRQSRKRLADMDFFETVDVETQKISDSLVDL
AMF_828 361 NILGNNRTLDHVMRRKLGIHEGDVYSTGAVRQSRKRLADMDFFETVDVETQKISDSLVDL
ACIS_00268 361 NIVGNNRTLDHVIRRKLGVYEGDVYSTGAVRQSRKRLADMDFFETVDVETRKVSDSLVDL


AM1096_6DE 421 NFRVKERGTGSFDIGAGFSSESGLVGKISVRERNAFGTGKMVAFDLSRSMTSLSGTLDLV
AM1096_Dawn 421 NFRVKERGTGSFDIGAGFSSESGLVGKISVRERNAFGTGKMVAFDLSRSMTSLSGTLDLV
AM1096_C51 421 NFRVKERGTGSFDIGAGFSSESGLVGKISVRERNAFGTGKMVAFDLSRSMTSLSGTLDLV
AM1096_C52 421 NFRVKERGTGSFDIGAGFSSESGLVGKISVRERNAFGTGKMVAFDLSRSMTSLSGTLDLV
AM1096_EMΦ 421 NFRVKERGTGSFDIGAGFSSESGLVGKISVRERNAFGTGKMVAFDLSRSMTSLSGTLDLV
AM1096_N4506 421 NFRVKERGTGSFDIGAGFSSESGLVGKISVRERNAFGTGKMVAFDLSRSMTSLSGTLDLV
AM1096_N3571 421 NFRVKERGTGSFDIGAGFSSESGLVGKISVRERNAFGTGKMVAFDLSRSMTSLSGTLDLV
AM1096_PR 421 NFRVKERGTGSFDIGAGFSSESGLVGKISVRERNAFGTGKMVAFDLSRSMTSLSGTLDLV
AM1096_VA 421 NFRVKERGTGSFDIGAGFSSESGLVGKISVRERNAFGTGKMVAFDLSRSMTSLSGTLDLV
AM1096_StM 421 NFRVKERGTGSFDIGAGFSSESGLVGKISVRERNAFGTGKMVAFDLSRSMTSLSGTLDLV
AMF_828 421 NFRVKERGTGSFDIGAGFSSESGLVGKISVRERNAFGTGKMVAFDLSRSMTSLSGTLDLV
ACIS_00268 421 NFKVKERGTGSFDIGAGFSSESGLVGKVSVRERNVLGTGKMIAFDLSRSMTSLSGTLDLV

AM1096_6DE 481 TPNVLDSDVAFGVGVFYSQQGSPS---SSSGTLGGLFSSSEGSFSSTNAGLSTRLSCNLT
AM1096_Dawn 481 TPNVLDSDVAFGVGVFYSQQGSPS---SSSGTLGGLFSSSEGSFSSTNAGLSTRLSCNLT
AM1096_C51 481 TPNVLDSDVAFGVGVFYSQQGSPS---SSSGTLGGLFSSSEGSFSSTNAGLSTRLSCNLT
AM1096_C52 481 TPNVLDSDVAFGVGVFYSQQGSPS---SSSGTLGGLFSSSEGSFSSTNAGLSTRLSCNLT
AM1096_EMΦ 481 TPNVLDSDVAFGVGVFYSQQGSPS---SSSGTLGGLFSSSEGSFSSTNAGLSTRLSCNLT
AM1096_N4506 481 TPNVLDSDVAFGVGVFYSQQGSPS---SSSGTLGGLFSSSEGSFSSTNAGLSTRLSCNLT
AM1096_N3571 481 TPNVLDSDVAFGVGVFYSQQGSPS---SSSGTLGGLFSSSEGSFSSTNAGLSTRLSCNLT
AM1096_PR 481 TPNVLDSDVAFGVGVFYSQQGSPS---SSSGTLGGLFSSSEGSFSSTNAGLSTRLSCNLT
AM1096_VA 481 TPNVLDSDVAFGVGVFYSQQGSPS---SSSGTLGGLFSSSEGSFSSTNAGLSTRLSCNLT
AM1096_StM 481 TPNVLDSDVAFGVGVFYSQQGSPS---SSSGTLGGLFSSSEGSFSSTNAGLSTRLSCNLT
AMF_828 481 TPNVLDSDVAFGVGVFYSQQGSPS---SSSGTLGGLFSSSEGSFSSTNAGLSTRLSCNLT
ACIS_00268 481 TPNVFDSDVAFGMGVFYSRQGSSSPAGSGGGVFGGLLPSSEGSFSSTNAGLSTRLSCSLT


AM1096_6DE 538 DSVAASLQYYYKYHSIHNIGESASIYIKEQEGRHFDSAVGYSLVYSSLDSTYKPSTGVFA
AM1096_Dawn 538 DSVAASLQYYYKYHSIHNIGESASIYIKEQEGRHFDSAVGYSLVYSSLDSTYKPSTGVFA
AM1096_C51 538 DSVAASLQYYYKYHSIHNIGESASIYIKEQEGRHFDSAVGYSLVYSSLDSTYKPSTGVFA
AM1096_C52 538 DSVAASLQYYYKYHSIHNIGESASIYIKEQEGRHFDSAVGYSLVYSSLDSTYKPSTGVFA
AM1096_EMΦ 538 DSVAASLQYYYKYHSIHNIGESASIYIKEQEGRHFDSAVGYSLVYSSLDSTYKPSTGVFA
AM1096_N4506 538 DSVAASLQYYYKYHSIHNIGESASIYIKEQEGRHFDSAVGYSLVYSSLDSTYKPSTGVFA
AM1096_N3571 538 DSVAASLQYYYKYHSIHNIGESASIYIKEQEGRHFDSAVGYSLVYSSLDSTYKPSTGVFA
AM1096_PR 538 DSVAASLQYYYKYHSIHNIGESASIYIKEQEGRHFDSAVGYSLVYSSLDSTYKPSTGVFA
AM1096_VA 538 DSVAASLQYYYKYHSIHNIGESASIYIKEQEGRHFDSAVGYSLVYSSLDSTYKPSTGVFA
AM1096_StM 538 DSVAASLQYYYKYHSIHNIGESASIYIKEQEGRHFDSAVGYSLVYSSLDSTYKPSTGVFA
AMF_828 538 DSVAASLQYYYKYHSIHNIGESASIYIKEQEGRHFDSAVGYSLVYSSLDSTYKPSTGVFA
ACIS_00268 541 DSVATSLQYSYKYHSIHNVGASASTYIKEQEGRHLDSAIGYSLVYSNLDSVYRPSRGVFA

AM1096_6DE 598 KVSQLFSGIGGNLHYVKTEASSAHFFPVFRRIHDDIVLKVKPSFGYVFAYSGETVKIGQR
AM1096_Dawn 598 KVSQLFSGIGGNLHYVKTEASSSHFFPVFRRIHDDIVLKVKPSFGYVFAYSGETVKIGQR
AM1096_C51 598 KVSQLFSGIGGNLHYVKTEASSAHFFPVFRRIHDDIVLKVKPSFGYVFAYSGETVKIGQR
AM1096_C52 598 KVSQLFSGIGGNLHYVKTEASSAHFFPVFRRIHDDIVLKVKPSFGYVFAYSGETVKIGQR
AM1096_EMΦ 598 KVSQLFSGIGGNLHYVKTEASSAHFFPVFRRIHDDIVLKVKPSFGYVFAYSGETVKIGQR
AM1096_N4506 598 KVSQLFSGIGGNLHYVKTEASSSHFFPVFRRIHDDIVLKVKPSFGYVFAYSGETVKIGQR
AM1096_N3571 598 KVSQLFSGIGGNLHYVKTEASSSHFFPVFRRIHDDIVLKVKPSFGYVFAYSGETVKIGQR
AM1096_PR 598 KVSQLFSGIGGNLHYVKTEASSAHFFPVFRRIHDDIVLKVKPSFGYVFAYSGETVKIGQR
AM1096_VA 598 KVSQLFSGIGGNLHYVKTEASSAHFFPVFRRIHDDIVLKVKPSFGYVFAYSGETVKIGQR
AM1096_StM 598 KVSQLFSGIGGNLHYVKTEASSAHFFPVFRRIHDDIVLKVKPSFGYVFAYSGETVKIGQR
AMF_828 598 KVSQLFSGIGGNLHYVKTEASSAHFFPVFRRIHDDIVLKVKPSFGYVFAYSGETVKIGQR
ACIS_00268 601 KVSQSFSGIGGNLHYVKTEASSAHFFPVFRRIHSDIVLKIKPSFGYVFAYLGETVKIGQR


AM1096_6DE 658 FFAGNSEIRGFAASGIGPRDRNTKESLGGKLFYGVVTQLDFPIGLPEHLGIRGSVFADVA
AM1096_Dawn 658 FFAGNSEIRGFAASGIGPRDRNTKESLGGKLFYGVVTQLDFPIGLPEHLGIRGSVFADVA
AM1096_C51 658 FFAGNSEIRGFAASGIGPRDRNTKESLGGKLFYGVVTQLDFPIGLPEHLGIRGSVFADVA
AM1096_C52 658 FFAGNSEIRGFAASGIGPRDRNTKESLGGKLFYGVVTQLDFPIGLPEHLGIRGSVFADVA
AM1096_EMΦ 658 FFAGNSEIRGFAASGIGPRDRNTKESLGGKLFYGVVTQLDFPIGLPEHLGIRGSVFADVA
AM1096_N4506 658 FFAGNSEIRGFAASGIGPRDRNTKESLGGKLFYGVVTQLDFPIGLPEHLGIRGSVFADVA
AM1096_N3571 658 FFAGNSEIRGFAASGIGPRDRNTKESLGGKLFYGVVTQLDFPIGLPEHLGIRGSVFADVA
AM1096_PR 658 FFAGNSEIRGFAASGIGPRDRNTKESLGGKLFYGVVTQLDFPIGLPEHLGIRGSVFADVA
AM1096_VA 658 FFAGNSEIRGFAASGIGPRDRNTKESLGGKLFYGVVTQLDFPIGLPEHLGIRGSVFADVA
AM1096_StM 658 FFAGNSEIRGFAASGIGPRDRNTKESLGGKLFYGVVTQLDFPIGLPEHLGIRGSVFADVA
AMF_828 658 FFAGNSEIRGFAASGIGPRDRNTKESLGGKLFYGVVTQLDFPIGLPEHLGIRGSVFADVA
ACIS_00268 661 FFAGNSEIRGFAASGIGPRDRTTKESLGGKLFYGVTAQFDFPIGLPEHLGIRGSVFADVA

AM1096_6DE 718 SLSRLDSEVGGYDTSDLPRLSIGFGFSWKSPFGPVRIDFGFPIVKEKFDIKDRIRISTDA
AM1096_Dawn 718 SLSRLDSEVGGYDTSDLPRLSIGFGFSWKSPFGPVRIDFGFPIVKEKFDIKDRIRISTDA
AM1096_C51 718 SLSRLDSEVGGYDTSDLPRLSIGFGFSWKSPFGPVRIDFGFPIVKEKFDIKDRIRISTDA
AM1096_C52 718 SLSRLDSEVGGYDTSDLPRLSIGFGFSWKSPFGPVRIDFGFPIVKEKFDIKDRIRISTDA
AM1096_EMΦ 718 SLSRLDSEVGGYDTSDLPRLSIGFGFSWKSPFGPVRIDFGFPIVKEKFDIKDRIRISTDA
AM1096_N4506 718 SLSRLDSEVGGYDTSDLPRLSIGFGFSWKSPFGPVRIDFGFPIVKEKFDIKDRIRISTDA
AM1096_N3571 718 SLSRLDSEVGGYDTSDLPRLSIGFGFSWKSPFGPVRIDFGFPIVKEKFDIKDRIRISTDA
AM1096_PR 718 SLSRLDSEVGGYDTSDLPRLSIGFGFSWKSPFGPVRIDFGFPIVKEKFDIKDRIRISTDA
AM1096_VA 718 SLSRLDSEVGGYDTSDLPRLSIGFGFSWKSPFGPVRIDFGFPIVKEKFDIKDRIRISTDA
AM1096_StM 718 SLSRLDSEVGGYDTSDLPRLSIGFGFSWKSPFGPVRIDFGFPIVKEKFDIKDRIRISTDA
AMF_828 718 SLSRLDSEVGGYDTSDLPRLSIGFGFSWKSPFGPVRIDFGFPIVKEKFDIKDRIRISTDA
ACIS_00268 721 SLSRLDAKAGGYDTSDLPRLSVGFGFSWQSPFGPVRIDFGFPLVKEKFDIKDRIRISTDA


AM1096_6DE 778 GI
AM1096_Dawn 778 GI
AM1096_C51 778 GI
AM1096_C52 778 GI
AM1096_EMΦ 778 GI
AM1096_N4506 778 GI
AM1096_N3571 778 GI
AM1096_PR 778 GI
AM1096_VA 778 GI
AM1096_StM 778 GI
AMF_828 778 GI
ACIS_00268 781 GI


Fig. S6. Amino acid alignment of AM1096 for all *A. marginale* strains and isolates. AMF_828 is the Florida strain homolog of AM1096. ACIS_00268 is the *A. marginale* ss. *centrale* ortholog of AM1096.
