# Supplementary material for: Subdominant Outer Membrane Antigens in Anaplasma marginale: Conservation, Antigenicity, and Protective Capacity Using Recombinant Protein
Source: PLoS One. 2015 Jun 16;10(6):e0129309. doi: 10.1371/journal.pone.0129309 (PMC4469585; doi:10.1371/journal.pone.0129309)
Supplement: S7 Fig — The line above the alignment (AM854 aa19-68) indicates the binding domain of APH_0338 and denotes the peptide used in the ELISAs. Amino acids that share identity are in white text with a black background. Conservative amino acid replacements are in white text with a gray background. (PDF) [file pone.0129309.s007.pdf]

```
AM854       1 MLHRWLALCFLASFAVTGCGLF---SKEKVGMDIVGVPFS---AGRVEKVYFDFNKYEIK
APH_0338    1 MLRRSSFFCLLALLSVTSCGTLLPDSNVGVGRHDLGSHRSVAFAKKVEKVYFDIGKYDLK

AM854      55 GSGKKVLLGLVERMKADKRSTLLIIGHTDSRGTEEYNLALGERRANAVKEFILGCDRSLS
APH_0338   61 GPGKKVILELVEQLRQDDSMYLVVIGHADATGTEEYSLALGEKRANAVKQFIIGCDKSLA

AM854     115 PRISTQSRGKAEPEVLVYSSDFKEAEKAHAQNRRVVLIVECQHSVSPKKKMAIKWPFSFG
APH_0338  121 PRVTTQSRGKAEPEVLVYSTDAQEVEKANAQNRRAVIVVEFAH-----------------

AM854     175 RSAAKQDDVGSSEVSDEN-PVDDSSEGIASEEAAPEEGVVSEEAAEEAPEVAQDSSAGVV
APH_0338  180 ---------IPRSGVADMHAPVASSITSENSNASAEGEDM---EASEFSSAIAN-------

AM854     234 APE
APH_0338      ---
```

Figure S7. A global pairwise alignment of AM854 and APH_0338 (OmpA) using lalign. The line above the alignment (AM854 aa19-68) includes the binding domain of APH_0338 (aa59-74) and denotes the peptide used in the ELISAs. Amino acids that share identity are in white text with a black background. Conservative amino acid replacements are in white text with a gray background.
