# Supplementary material for: Subdominant Outer Membrane Antigens in Anaplasma marginale: Conservation, Antigenicity, and Protective Capacity Using Recombinant Protein
Source: PLoS One. 2015 Jun 16;10(6):e0129309. doi: 10.1371/journal.pone.0129309 (PMC4469585; doi:10.1371/journal.pone.0129309)
Supplement: S8 Fig — The line above the alignment (AM936 aa84-121) indicates the binding domain of APH_0248 and denotes the peptide used in the ELISAs. Amino acids that share identity are in white text with a black background. Conservative amino acid replacements are in white text with a gray background. (PDF) [file pone.0129309.s008.pdf]

```
AM936       1 M--------------SGEDEYKEIIRQCIGSVKEVFGEGRFDDVVASIMKMQEKVLASSM
APH_0248    1 MIPLAPWKSISVVYMSGSDEYKEIIKQCIGSVKEVFGEGRFDDVVASIMKMQEKVLASSM

AM936      47 KDGDP--VGQI-AADGVGNELYDRIADRLEERVSQKISEDLRIIKKRLLRLERVVLGGGS
APH_0248   61 QQDDTGTVGQIESGEGSGARLSDEQVQQLMNSIREEFKDDLRAIKRRILKLERAVYGA--

AM936     104 VSGDAAAHQVSGNQPSQQNSSAAAEGG
APH_0248  121 ------------NTPKES---------
```

Figure S8. A global pairwise alignment of AM936 and APH_0248 (Asp14) using lalign. The line above the alignment (AM936 aa84-121) includes the binding domain of APH_0248 (aa113-124) and denotes the peptide used in the ELISAs. Amino acids that share identity are in white text with a black background. Conservative amino acid replacements are in white text with a gray background.
