# Supplementary figures and images for: Subdominant Outer Membrane Antigens in Anaplasma marginale: Conservation, Antigenicity, and Protective Capacity Using Recombinant Protein
Source: PLoS One. 2015 Jun 16;10(6):e0129309. doi: 10.1371/journal.pone.0129309 (PMC4469585; doi:10.1371/journal.pone.0129309)

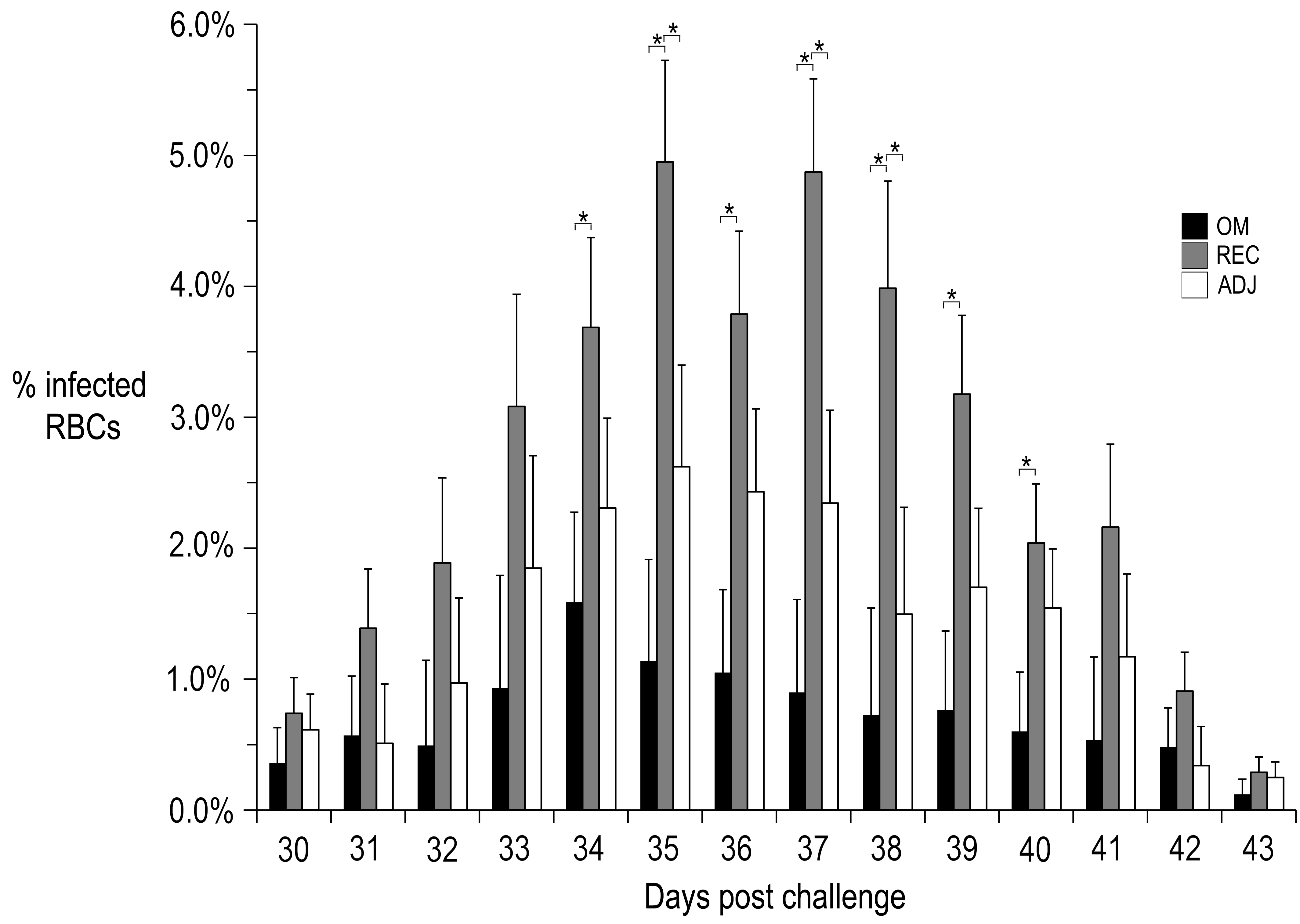

Supplement: S9 Fig — Each bar represents the mean of the percent infected erythrocytes for each group of animals for days 30 to 43 of challenge. The black bars represent the outer membrane (OM) vaccinates, the gray bars represent AM854/AM936 vaccinates (REC), and the white bars represent the adjuvant only vaccinates (ADJ). The error bars are the standard error of the mean. Brackets over the bars indicate groups that are statistically significantly different. The asterisk indicates a p value of <0.05. (TIF) [file pone.0129309.s009.tif]

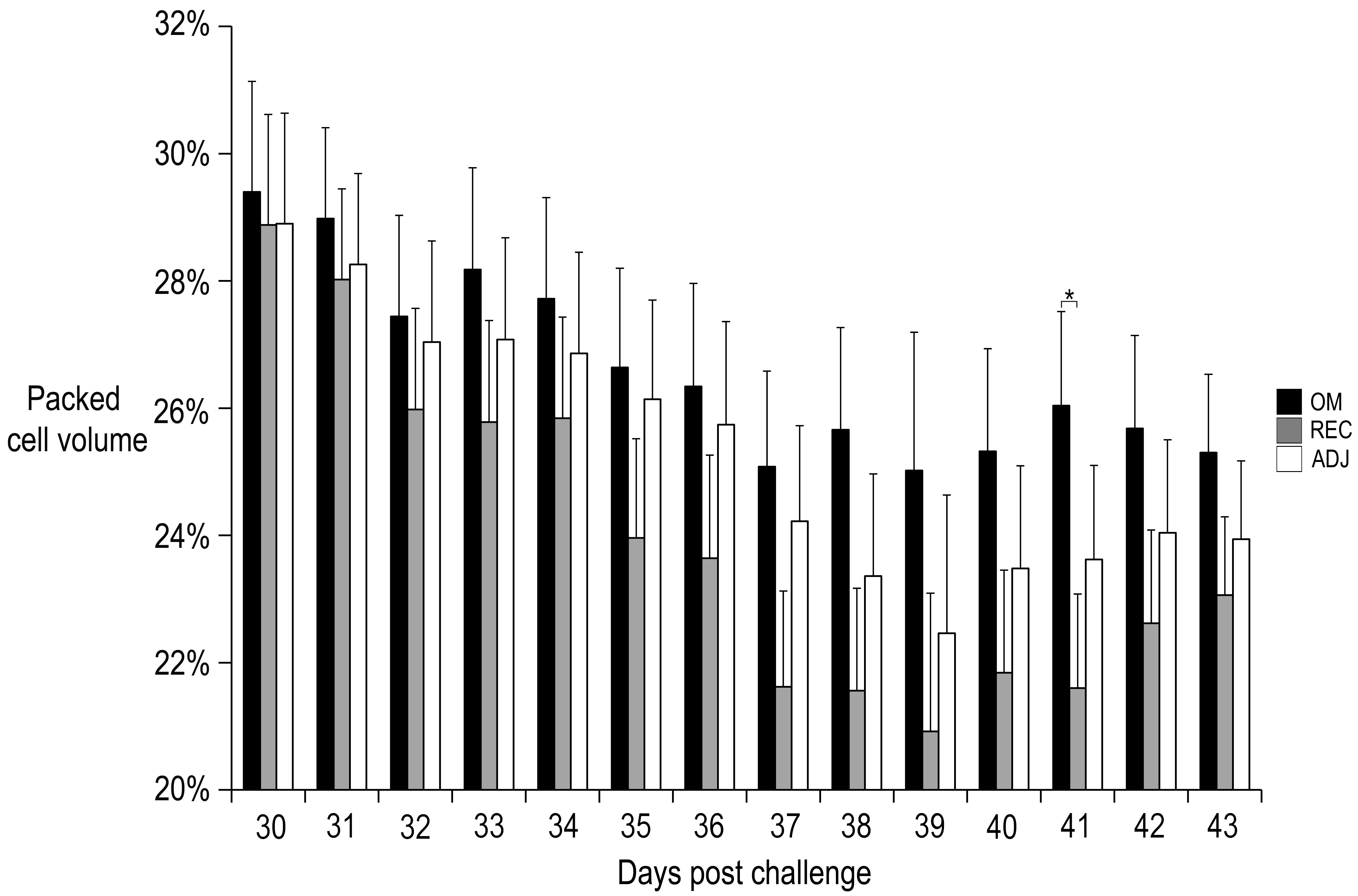

Supplement: S10 Fig — Each bar represents the mean of the PCV for each group of animals for days 30 to 42 of challenge. The black bars represent the outer membrane (OM) vaccinates, the gray bars represent AM854/AM936 vaccinates (REC), and the white bars represent the adjuvant only vaccinates (ADJ). The error bars are the standard error of the mean. Brackets over the bars indicate groups that are statistically significantly different. The asterisk indicates a p value of <0.05. (TIF) [file pone.0129309.s010.tif]
