# Supplementary material for: Subdominant Outer Membrane Antigens in Anaplasma marginale: Conservation, Antigenicity, and Protective Capacity Using Recombinant Protein
Source: PLoS One. 2015 Jun 16;10(6):e0129309. doi: 10.1371/journal.pone.0129309 (PMC4469585; doi:10.1371/journal.pone.0129309)
Supplement: S1 Table — (DOCX) [file pone.0129309.s011.docx]

Table S1. Oligonucleotides used for sequencing candidate genes.

| Locus Tag | Name | Oligonucleotide sequence |
| --- | --- | --- |
| AM202 | ForwardA | 5’GTTGGAGTCTATCACGCTTGC3’ |
|  | ReverseB | 5’CTGCAAGTTGCTTTGCGTAC3’ |
| AM368 | ForwardA | 5’GTAAAACGACGGCCAG3’ |
|  | FowardB | 5’TGGTGGGTGCTTGTTGCG3’ |
|  | ForwardC | 5’GCCCAGGATTAGATGTTCAGC3’ |
|  | ForwardD | 5’GCATCATCGCGCAAAGACAA3’ |
|  | ForwardE | 5’TGCTGAACATCTAATCCTGGG3’ |
|  | ReverseA | 5’CAGGAAACACCTATGAC3’ |
|  | ReverseB | 5’CGATGACAACAAGGCAGCACA3’ |
|  | ReverseC | 5’GCGGAAAATGCGGATACTCA3’ |
|  | ReverseD | 5’GCTCCTCCGAATCTGGTGTTATGTC3’ |
|  | ReverseE | 5’GCCATCGCTGTGCCTTATTG3’ |
| AM854 | Forward | 5’GTAAAACGACGGCCAG3’ |
|  | Reverse | 5’CAGGAAACACCTATGAC3’ |
| AM936 | Forward | 5’GGGAGGTTTTTACTATGCTGC3’ |
|  | Reverse | 5’GTGGTCGCGCCTGAATAGTA3’ |
| AM1041 | ForwardA | 5’GTAAAACGACGGCCAG3’ |
|  | FowardB | 5’ACTTTCACCGCTCTTGCCACT3’ |
|  | ReverseA | 5’CAGGAAACACCTATGAC3’ |
|  | ReverseB | 5’TGATGCCAGTGGTGTGTGTTG3’ |
| AM1096 | ForwardA | 5’GTAAAACGACGGCCAG3’ |
|  | ForwardB | 5'CTTGACTTCAGAGTCAAGTCC3' |
|  | ForwardC | 5'TTGACATAGGTGCTGGCTT3' |
|  | ForwardD | 5'GGGATAGGAGGAAACTTACAC3' |
|  | ForwardE | 5'CAGCCCATCTTCTTCCAGT3' |
|  | ForwardF | 5'GCTGGGTGCAAAGGTGTCTTA3' |
|  | ForwardG | 5'GGCATACATGAGGGAGACGT3' |
|  | ReverseA | 5’CAGGAAACACCTATGAC3’ |
|  | ReverseB | 5'GCTGACTCCCCTATGTTATGT3' |
|  | ReverseC | 5'CCCTGCACGTCATAGTCAC3' |
|  | ReverseD | 5'CCCTCTACAATGTTGAGCACT3' |
|  | ReverseE | 5'CCTCTGCCCTATTTTCACAGT3' |
|  | ReverseF | 5'AGTCATGCGCGTGAGGT3' |
|  | ReverseG | 5'GCGACCATCTTTCCAGTGCC3' |
