# Supplementary material for: Subdominant Outer Membrane Antigens in Anaplasma marginale: Conservation, Antigenicity, and Protective Capacity Using Recombinant Protein
Source: PLoS One. 2015 Jun 16;10(6):e0129309. doi: 10.1371/journal.pone.0129309 (PMC4469585; doi:10.1371/journal.pone.0129309)
Supplement: S2 Table — (DOCX) [file pone.0129309.s012.docx]

Table S2. Oligonucleotides used for PCR amplification for cloning and protein expression.

| Locus Tag | Name | Oligonucleotide Sequence |  |
| --- | --- | --- | --- |
| AM202 | Forward | 5'CAGGAGCGTTCTGTCGGC3' | |
|  | Reverse | 5'CTGCAAGTTGCTTTGCGTAC3' | |
| AM854 | Forward | 5'CACCCTTTTCAGCAAGGAAAAGGTCGGGA3' | |
|  | Reverse | 5'TTCAGGCGCGACCAC3' | |
| AM936 | Forward | 5'CACCATGTCTGGTGAAGACGAATA3' | |
|  | Reverse | 5'CCCACCTTCAGCAGCGG3' | |
| AM1096 | Forward | 5'CACCTTTGTGGGGCCCATTC3' | |
|  | Reverse | 5'AATACCCGCATCCGTGGAAATC3' | |
