# Supplementary material for: Subdominant Outer Membrane Antigens in Anaplasma marginale: Conservation, Antigenicity, and Protective Capacity Using Recombinant Protein
Source: PLoS One. 2015 Jun 16;10(6):e0129309. doi: 10.1371/journal.pone.0129309 (PMC4469585; doi:10.1371/journal.pone.0129309)
Supplement: S3 Table — (DOCX) [file pone.0129309.s013.docx]

Table S3. Pairwise amino acid identity among all isolates and strains for AM202.

| **AM202** | 6DE | Dawn | C51 | C52 | EMΦ | N3518 | N3571 | PR | VA | StM | AMF149 | ACIS 01081 |
| --- | --- | --- | --- | --- | --- | --- | --- | --- | --- | --- | --- | --- |
| 6DE | **100.0** |  |  |  |  |  |  |  |  |  |  |  |
| Dawn | 100.0 | **100.0** |  |  |  |  |  |  |  |  |  |  |
| C51 | 100.0 | 100.0 | **100.0** |  |  |  |  |  |  |  |  |  |
| C52 | 100.0 | 100.0 | 100.0 | **100.0** |  |  |  |  |  |  |  |  |
| EMΦ | 100.0 | 100.0 | 100.0 | 100.0 | **100.0** |  |  |  |  |  |  |  |
| N3518 | 100.0 | 100.0 | 100.0 | 100.0 | 100.0 | **100.0** |  |  |  |  |  |  |
| N3571 | 100.0 | 100.0 | 100.0 | 100.0 | 100.0 | 100.0 | **100.0** |  |  |  |  |  |
| PR | 100.0 | 100.0 | 100.0 | 100.0 | 100.0 | 100.0 | 100.0 | **100.0** |  |  |  |  |
| VA | 100.0 | 100.0 | 100.0 | 100.0 | 100.0 | 100.0 | 100.0 | 100.0 | **100.0** |  |  |  |
| StM | 100.0 | 100.0 | 100.0 | 100.0 | 100.0 | 100.0 | 100.0 | 100.0 | 100.0 | **100.0** |  |  |
| AMF149^a^ | 100.0 | 100.0 | 100.0 | 100.0 | 100.0 | 100.0 | 100.0 | 100.0 | 100.0 | 100.0 | **100.0** |  |
| ACIS 01081^b^ | 99.0 | 99.0 | 99.0 | 99.0 | 99.0 | 99.0 | 99.0 | 99.0 | 99.0 | 99.0 | 99.0 | **100.0** |

a. AMF149 is the homolog to AM202 in the Florida strain.

b. ACIS 01081 is the ortholog of AM202 in *A. marginale* ss. *centrale*.
