# Supplementary material for: Subdominant Outer Membrane Antigens in Anaplasma marginale: Conservation, Antigenicity, and Protective Capacity Using Recombinant Protein
Source: PLoS One. 2015 Jun 16;10(6):e0129309. doi: 10.1371/journal.pone.0129309 (PMC4469585; doi:10.1371/journal.pone.0129309)
Supplement: S4 Table — (DOCX) [file pone.0129309.s014.docx]

Table S4. Pairwise amino acid identity among all isolates and strains for AM368.

| **AM368** | 6DE | Dawn | C51 | C52 | EMΦ | N3518 | N3571 | PR | VA | StM | AMF 269 | ACIS 00938 |
| --- | --- | --- | --- | --- | --- | --- | --- | --- | --- | --- | --- | --- |
| 6DE | **100.0** |  |  |  |  |  |  |  |  |  |  |  |
| Dawn | 98.3 | **100.0** |  |  |  |  |  |  |  |  |  |  |
| C51 | 98.4 | 99.0 | **100.0** |  |  |  |  |  |  |  |  |  |
| C52 | 99.3 | 98.2 | 98.5 | **100.0** |  |  |  |  |  |  |  |  |
| EMΦ | 98.9 | 98.3 | 98.4 | 98.9 | **100.0** |  |  |  |  |  |  |  |
| N3518 | 98.8 | 98.8 | 99.4 | 98.7 | 98.2 | **100.0** |  |  |  |  |  |  |
| N3571 | 97.9 | 98.9 | 99.1 | 97.8 | 97.9 | 98.7 | **100.0** |  |  |  |  |  |
| PR | 98.7 | 98.4 | 98.2 | 98.5 | 97.9 | 98.6 | 98.1 | **100.0** |  |  |  |  |
| VA | 98.8 | 99.1 | 99.0 | 98.9 | 99.0 | 98.6 | 98.7 | 98.3 | **100.0** |  |  |  |
| StM | 98.6 | 98.7 | 98.6 | 98.5 | 98.5 | 98.4 | 98.1 | 98.5 | 98.8 | **100.0** |  |  |
| AMF269^a^ | 98.7 | 99.0 | 98.9 | 98.8 | 98.9 | 98.5 | 98.6 | 98.2 | 99.9 | 98.8 | **100.0** |  |
| ACIS00938^b^ | 38.2 | 38.2 | 38.3 | 38.3 | 38.1 | 38.3 | 38.0 | 38.3 | 38.2 | 38.3 | 38.2 | **100.0** |

^a.^ AMF269 is the homolog to AM368 in the Florida strain.

^b.^ ACIS 00938 is the ortholog of AM368 in *A. marginale* ss. *centrale.*
