# Supplementary material for: Subdominant Outer Membrane Antigens in Anaplasma marginale: Conservation, Antigenicity, and Protective Capacity Using Recombinant Protein
Source: PLoS One. 2015 Jun 16;10(6):e0129309. doi: 10.1371/journal.pone.0129309 (PMC4469585; doi:10.1371/journal.pone.0129309)
Supplement: S5 Table — (DOCX) [file pone.0129309.s015.docx]

Table S5. Pairwise amino acid identity among all isolates and strains for AM854.

| **AM854** | 6DE | Dawn | C51 | C52 | EMΦ | N3574 | N4506 | PR | VA | StM | AMF640 | ACIS 00486 |
| --- | --- | --- | --- | --- | --- | --- | --- | --- | --- | --- | --- | --- |
| 6DE | **100.0** |  |  |  |  |  |  |  |  |  |  |  |
| Dawn | 100.0 | **100.0** |  |  |  |  |  |  |  |  |  |  |
| C51 | 100.0 | 100.0 | **100.0** |  |  |  |  |  |  |  |  |  |
| C52 | 100.0 | 100.0 | 100.0 | **100.0** |  |  |  |  |  |  |  |  |
| EMΦ | 99.6 | 99.6 | 99.6 | 99.6 | **100.0** |  |  |  |  |  |  |  |
| N3574 | 100.0 | 100.0 | 100.0 | 100.0 | 99.6 | **100.0** |  |  |  |  |  |  |
| N4506 | 100.0 | 100.0 | 100.0 | 100.0 | 99.6 | 100.0 | **100.0** |  |  |  |  |  |
| PR | 100.0 | 100.0 | 100.0 | 100.0 | 99.6 | 100.0 | 100.0 | **100.0** |  |  |  |  |
| VA | 100.0 | 100.0 | 100.0 | 100.0 | 99.6 | 100.0 | 100.0 | 100.0 | **100.0** |  |  |  |
| StM | 100.0 | 100.0 | 100.0 | 100.0 | 99.6 | 100.0 | 100.0 | 100.0 | 100.0 | **100.0** |  |  |
| AMF640^a^ | 100.0 | 100.0 | 100.0 | 100.0 | 99.6 | 100.0 | 100.0 | 100.0 | 100.0 | 100.0 | **100.0** |  |
| ACIS 00486^b^ | 78.8 | 78.8 | 78.8 | 78.8 | 78.8 | 78.8 | 78.8 | 78.8 | 78.8 | 78.8 | 78.8 | **100.0** |

^a.^ AMF640 is the homolog to AM854 in the Florida strain.

^b.^ ACIS 00486 is the ortholog of AM854 in *A. marginale* ss. *centrale.*
