# Supplementary material for: Subdominant Outer Membrane Antigens in Anaplasma marginale: Conservation, Antigenicity, and Protective Capacity Using Recombinant Protein
Source: PLoS One. 2015 Jun 16;10(6):e0129309. doi: 10.1371/journal.pone.0129309 (PMC4469585; doi:10.1371/journal.pone.0129309)
Supplement: S7 Table — (DOCX) [file pone.0129309.s017.docx]

Table S7. Pairwise amino acid identity among all isolates and strains for AM1041.

| **AM1041** | 6DE | Dawn | C51 | C52 | EMΦ | N3518.1 | N3518.2 | N3571 | PR | VA.1 | VA.2 | StM | AMF790 | ACIS 00314 |
| --- | --- | --- | --- | --- | --- | --- | --- | --- | --- | --- | --- | --- | --- | --- |
| 6DE | **100.0** |  |  |  |  |  |  |  |  |  |  |  |  |  |
| Dawn | 97.8 | **100.0** |  |  |  |  |  |  |  |  |  |  |  |  |
| C51 | 99.4 | 98.2 | **100.0** |  |  |  |  |  |  |  |  |  |  |  |
| C52 | 99.2 | 98.0 | 99.0 | **100.0** |  |  |  |  |  |  |  |  |  |  |
| EMΦ | 99.0 | 97.1 | 98.4 | 98.2 | **100.0** |  |  |  |  |  |  |  |  |  |
| N3518.1^a^ | 98.0 | 99.6 | 98.6 | 97.6 | 97.4 | **100.0** |  |  |  |  |  |  |  |  |
| N3518.2^a^ | 98.2 | 99.6 | 98.6 | 97.5 | 97.5 | 100.0 | **100.0** |  |  |  |  |  |  |  |
| N3571 | 97.8 | 99.2 | 98.2 | 97.1 | 97.1 | 99.6 | 99.6 | **100.0** |  |  |  |  |  |  |
| PR | 100.0 | 97.8 | 99.4 | 99.2 | 99.0 | 98.0 | 98.2 | 97.8 | **100.0** |  |  |  |  |  |
| VA.1^b^ | 100.0 | 97.8 | 99.4 | 99.2 | 99.0 | 98.0 | 98.2 | 97.8 | 100.0 | **100.0** |  |  |  |  |
| VA.2^b^ | 98.4 | 98.2 | 98.6 | 98.4 | 98.6 | 97.6 | 97.7 | 97.3 | 98.4 | 98.4 | **100.0** |  |  |  |
| StM | 99.4 | 98.2 | 100.0 | 99.0 | 98.4 | 98.6 | 98.6 | 98.2 | 99.4 | 99.4 | 98.4 | **100.0** |  |  |
| AMF790^c^ | 100.0 | 97.8 | 99.4 | 99.2 | 99.0 | 98.0 | 98.2 | 97.8 | 100.0 | 100.0 | 98.2 | 99.4 | **100.0** |  |
| ACIS 00314^d^ | 98.8 | 96.9 | 98.2 | 98.0 | 99.0 | 97.1 | 97.3 | 96.9 | 98.8 | 98.8 | 98.6 | 98.2 | 98.8 | **100.0** |

^a.^ N3518.1 and N3518.2 are variants that were obtained from the same isolate from Nayarit, Mexico.

^b.^ VA.1 and VA.2 are variants that were obtained from the Virginia strain.

^c.^ AMF790 is the homolog to AM1041 in the Florida strain.

^d.^ ACIS 00314 is the ortholog of AM1041 in *A. marginale* ss. *Centrale*.
