# Supplementary material for: Subdominant Outer Membrane Antigens in Anaplasma marginale: Conservation, Antigenicity, and Protective Capacity Using Recombinant Protein
Source: PLoS One. 2015 Jun 16;10(6):e0129309. doi: 10.1371/journal.pone.0129309 (PMC4469585; doi:10.1371/journal.pone.0129309)
Supplement: S8 Table — (DOCX) [file pone.0129309.s018.docx]

Table S8. Pairwise amino acid identity among all isolates and strains for AM1096.

| **AM1096** | 6DE | | AU | | C51 | | C52 | | EMΦ | | N3571 | | N4506 | | PR | | VA | | StM | | AMF 828 | | ACIS 00268 | |
| --- | --- | --- | --- | --- | --- | --- | --- | --- | --- | --- | --- | --- | --- | --- | --- | --- | --- | --- | --- | --- | --- | --- | --- | --- |
| 6DE | | **100.0** | |  | |  | |  | |  | |  | |  | |  | |  | |  | |  | |  |
| Dawn | 99.7 | | **100.0** | |  | |  | |  | |  | |  | |  | |  | |  | |  | |  | |
| C51 | 100.0 | | 99.7 | | **100.0** | |  | |  | |  | |  | |  | |  | |  | |  | |  | |
| C52 | 100.0 | | 99.7 | | 100.0 | | **100.0** | |  | |  | |  | |  | |  | |  | |  | |  | |
| EMΦ | 99.9 | | 99.6 | | 99.9 | | 99.9 | | **100.0** | |  | |  | |  | |  | |  | |  | |  | |
| N3571 | 99.7 | | 100.0 | | 99.7 | | 99.7 | | 99.6 | | **100.0** | |  | |  | |  | |  | |  | |  | |
| N4506 | 99.9 | | 99.9 | | 99.9 | | 99.9 | | 99.7 | | 99.9 | | **100.0** | |  | |  | |  | |  | |  | |
| PR | 100.0 | | 99.7 | | 100.0 | | 100.0 | | 99.9 | | 99.7 | | 99.9 | | **100.0** | |  | |  | |  | |  | |
| VA | 100.0 | | 99.7 | | 100.0 | | 100.0 | | 99.9 | | 99.7 | | 99.9 | | 100.0 | | **100.0** | |  | |  | |  | |
| StM | 100.0 | | 99.7 | | 100.0 | | 100.0 | | 99.9 | | 99.7 | | 99.9 | | 100.0 | | 100.0 | | **100.0** | |  | |  | |
| AMF828^a^ | 100.0 | | 99.7 | | 100.0 | | 100.0 | | 99.9 | | 99.7 | | 99.9 | | 100.0 | | 100.0 | | 100.0 | | **100.0** | |  | |
| ACIS 00268^b^ | 87.7 | | 87.9 | | 87.7 | | 87.7 | | 87.9 | | 87.9 | | 87.7 | | 87.7 | | 87.7 | | 87.7 | | 87.7 | | **100.0** | |

^a.^ AMF828 is the homolog to AM1096 in the Florida strain.

^b.^ ACIS 00268 is the ortholog of AM1096 in *A. marginale* ss. *centrale*.
